# Global trends and health inequalities in mental disorders among older adults: a comprehensive analysis using global burden of disease 2021 data

**DOI:** 10.3389/fpubh.2025.1644610

**Published:** 2025-08-26

**Authors:** Ying Chen, Xiao Dong, Wenxing Xu, Yudan Long, Kai Liu

**Affiliations:** ^1^Medical Laboratory Center, Hainan General Hospital, Hainan Affiliated Hospital of Hainan Medical University, Hainan Medical University, Haikou, Hainan, China; ^2^Geriatric Center, Hainan General Hospital, Hainan Affiliated Hospital of Hainan Medical University, Hainan Medical University, Haikou, Hainan, China; ^3^Hainan Medical University, Haikou, Hainan, China

**Keywords:** geriatric mental health, global burden of disease, socio-demographic index (SDI), disability-adjusted life years (DALYs), health inequalities

## Abstract

**Objective:**

This study aims to analyze global trends and health inequalities in mental disorders among older adults (≥60 years) using Global Burden of Disease (GBD) 2021 data, with a focus on informing equity-focused public health strategies to address disparities in burden and access to care.

**Methods:**

Using GBD 2021 data, we examined mental disorders in adults ≥60 across 204 countries (1990–2021). We calculated age-standardized incidence (ASIR) and disability-adjusted life years (DALYs) rates and estimated annual percentage change (EAPC). Bayesian Age-Period-Cohort modeling and frontier analysis assessed trends and projections. Analyses used R (v4.4.2) and JD_GBDR software.

**Findings:**

In 2021, there were 74.9 million (95%UI: 59.8–94.2) new mental disorder cases among older adults globally, with an ASIR of 6,867.6 per 100,000. ASIR remained stable since 1990 (EAPC: 0.01), but varied regionally from 13,024.1 (Central Sub-Saharan Africa) to 4,643.9 (Oceania). The age-standardized DALY rate (ASDR) was 2,095.4 per 100,000, highest in low-SDI regions (2,423.9) and lowest in high-SDI regions (1,934.2). Females had higher ASIR (7,483.7 vs. 5,181.6) and ASDR (2,198.3 vs. 1,760.2) than males, with male DALYs increasing (EAPC: 0.07). By 2035, ASDR is projected to rise to 2,494.5, with depressive and anxiety disorders predominant in high-SDI regions and schizophrenia more prevalent in low-SDI areas.

**Conclusion:**

Significant disparities persist, with low-SDI regions and women disproportionately affected. Targeted strategies should strengthen mental healthcare access, implement sex-specific interventions, and address aging-related challenges.

## 1 Introduction

The rapid growth of the global aging population—driven by improved healthcare, declining mortality, and increased life expectancy—has intensified the burden of mental disorders among older adults (≥60 years), including depression, anxiety disorders, schizophrenia, and bipolar disorder (1). Notably, insomnia and sleep disturbances, which affect up to 50% of older adults, are closely linked to depression and cognitive decline, further exacerbating the disease burden (2, 3). National surveys reveal stark disparities: while high-income countries report a 15–25% prevalence of late-life depression, LMICs show higher rates (e.g., 30% in sub-Saharan Africa) due to limited healthcare access (4). Mental health in older adults is uniquely challenging due to underreporting (stemming from stigma), misattribution of symptoms to “normal aging,” and high comorbidity with chronic diseases (5). These conditions severely impair cognitive function [e.g., executive dysfunction (6)], emotional regulation, and quality of life, while increasing dependency rates by 2–3-fold (7). The economic toll is staggering, with dementia-related costs projected to exceed USD 2 trillion by 2030 (8).

Despite their significant impact, mental disorders in older adults remain underreported, especially in LMICs, where limited healthcare access and diagnostic challenges persist. For example, fewer than 10% of older adults with depression in sub-Saharan Africa receive treatment, compared to 30–50% in high-income regions (9). Gender disparities further complicate the issue: women face higher stigma-related barriers, while men are often misdiagnosed due to atypical symptoms (10). These gaps highlight the need for standardized, data-driven approaches to address inequities in mental healthcare for aging populations.

Using the comprehensive GBD 2021 dataset, this study provides age-, sex-, and SDI-stratified estimates of mental disorder burden across 204 countries. Mental disorders were classified according to GBD 2021 case definitions, which align with ICD-10 diagnostic criteria and incorporate clinical prevalence studies, hospital records, and systematic reviews. To address underreporting in LMICs, we applied GBD’s standardized case ascertainment corrections and disability weights, which account for differences in healthcare access and diagnostic practices across settings. Our analysis leverages frontier modeling and Bayesian projections to identify high-risk populations and subtype-specific trends (e.g., schizophrenia in low-SDI regions vs. anxiety disorders in high-SDI areas). These insights aim to inform targeted interventions, from community-based care in LMICs to prevention strategies in aging societies, aligning with global healthy aging goals.

## 2 Materials and methods

### 2.1 Data sources

Since 1990, the Global Burden of Disease (GBD) database has systematically collected and integrated epidemiological data from 21 GBD regions and 204 countries and territories (11). The GBD 2021 study provides the most recent analysis of epidemiological data for 371 diseases and injuries and 88 risk factors (12). All required data were extracted from the Global Health Data Exchange (GHDx) query tool.^1^ Our dataset included sex-, age-, incidence-, and disability-adjusted life year (DALY)-specific estimates related to mental disorders across 21 GBD regions and 204 countries and territories.

### 2.2 Health inequality analysis

The SDI is a composite indicator reflecting societal development levels and is strongly correlated with health outcomes (13). It is calculated as the geometric mean of three components: total fertility rate among individuals under 25 years, mean educational attainment among those aged 15 and older, and lag-distributed income per capita, scaled between 0 and 1 (14). In the GBD 2021 study, countries and territories were categorized into five SDI quintiles: low, low-middle, middle, high-middle, and high SDI. To assess temporal trends in health inequality, we compared data from 204 countries and territories between 1990 and 2021.

To mitigate bias and heterogeneity, we employed robust regression models (RLM) instead of ordinary linear regression (LM) for health inequality analyses. Robust regression reduces sensitivity to outliers, minimizes bias from data heterogeneity or extreme values, and provides a more accurate representation of health disparities (15). Additionally, the concentration index was calculated by matching cumulative proportions of incidence and DALYs with cumulative population distributions ranked by SDI, followed by numerical integration of the area under the Lorenz curve.

### 2.3 Frontier analysis

To evaluate the relationship between the burden of mental disorders in older adults and socio-demographic development, we constructed a frontier model using SDI as the predictor and ASIR and ASDR as outcomes. Unlike traditional regression models that describe variable relationships or predict outcomes, frontier analysis employs advanced statistical techniques to account for nonlinear associations between SDI and disease burden, capturing multidimensional drivers of mental disorder burden in aging populations.

The frontier approach identifies the theoretical minimum ASR achievable for each country or territory given its current SDI level, serving as a benchmark for optimal performance. This method quantifies the gap between a country’s current burden and its potential minimum burden, highlighting areas for improvement. The optimal performance boundary was fitted using locally weighted regression (LOESS) (smoothing span tested at 0.3–0.5 for sensitivity). To ensure robustness, we performed 1,000 bootstrap iterations to generate 95% uncertainty intervals (UIs) and computed the efficiency gap (absolute distance between observed ASDR and the frontier) as an indicator of improvement potential.

### 2.4 Bayesian age-period-cohort model projections

We applied the BAPC model to forecast future disease burden trends. This model extends the traditional generalized linear model (GLM) framework within a Bayesian framework, dynamically integrating age, period, and cohort (APC) effects. These effects were modeled as time-varying parameters smoothed via second-order random walks, improving posterior probability estimation. A key strength of the BAPC model is its use of integrated nested Laplace approximation (INLA) for approximating marginal posterior distributions (16). This approach avoids convergence and mixing challenges typical of Markov chain Monte Carlo (MCMC) methods while maintaining computational efficiency (17). The model’s flexibility in handling time-series data makes it particularly suitable for long-term burden projections (18). Due to its comprehensive coverage and ability to capture temporal trends, the BAPC model has been widely validated in epidemiological studies, particularly those involving age-structured populations and complex cohort effects (19). In this study, we implemented the BAPC model using the “BAPC” R package, leveraging GBD 2021 data and Institute for Health Metrics and Evaluation (IHME) population projections. This approach enabled nuanced predictions of future mental disorder burden in older adults, accounting for the complex interplay of age, period, and cohort effects.

### 2.5 Statistical analysis and data visualization

This study analyzed the burden of mental disorders among people over 60 years across age groups, sexes, years (1990–2021), and geographic locations using Global Burden of Disease (GBD) 2021 data. ASIR and ASDR per 100,000 population were calculated with 95% uncertainty intervals (UIs) using GBD’s standardized demographic methods. Temporal trends were assessed via EAPC, derived from a log-linear regression model: ln(ASR) = *α* + *β* × calendar year + *ε*, where EAPC = 100 × (exp(β) − 1). Trends were classified as increasing (EAPC and 95% confidence interval [CI] lower bound >0), decreasing (EAPC and 95% CI upper bound <0), or stable (95% CI spanning 0). Socioeconomic patterns were evaluated by stratifying countries into quintiles based on the SDI (SDI; range: 0–1). Correlation analyses (Pearson’s r) examined associations between ASR, SDI, and EAPC. Frontier analysis identified optimal achievable ASR-SDI relationships. All statistical analyses and data visualizations were performed using R (version 4.4.2) and JD_GBDR (V2.37, Jingding Medical Technology Co., Ltd.). In this study, the R software package (version 4.2.3) and JD_GBDR (V2.22, Jingding Medical Technology Co., Ltd.) was used for the drawing of the figures. Uncertainty was propagated throughout all calculations using 1,000 posterior draws from GBD’s cause-specific mortality and disability models.

## 3 Results

### 3.1 Global and regional patterns

**Figure 1:**
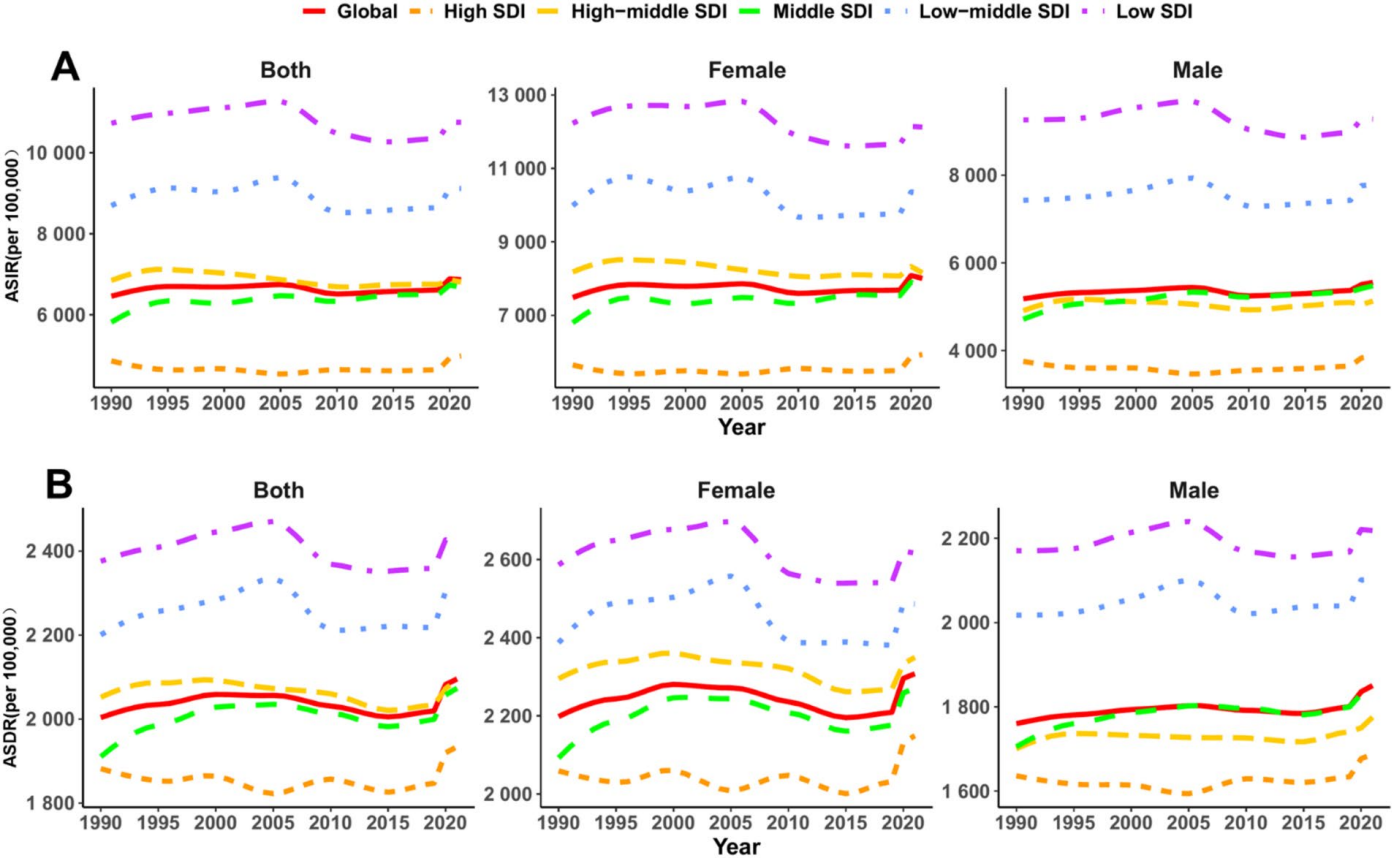
Temporal trends in sex-specific age-standardized incidence, DALY rates of mental disorders across socio-demographic index regions among people over 60 years from 1990 to 2021. **(A)** Age-standardized incidence rate (ASIR); **(B)** Age-standardized DALY rate (ASDR).

In 2021, there were 74.9 million (95% UI: 59.8–94.2 million) new cases of mental disorders among people over 60 years globally, with an ASIR of 6,867.63 per 100,000 population (95% UI: 5,470.33–8,645.08). Although the absolute number of cases increased significantly compared to 1990, the rise in ASIR was minimal (Table 1; Figure 1A). From 1990 to 2021, the EAPC in ASIR was 0.01 (95% CI: −0.05 to 0.07), indicating a stable trend.

**Table 1:** Age-standardized incidence rates and absolute case counts (in hundreds) of mental disorders among individuals aged ≥60 years from 1990 to 2021.

	1990		2021		EAPC (95%CI)
	Numbers (95%UI)	ASIR (95%UI)	Numbers (95%UI)	ASIR (95%UI)	
Global	312660.63 (390938.47–251465.38)	6456.95 (8083.8–5177.04)	749482.93 (942280.7–598222.88)	6867.63 (8645.08–5470.33)	0.01 (−0.05 to 0.07)
Sex					
Female	200214.14 (250463.85–160809.79)	7483.7 (9367.12–6004.6)	468265.54 (589041.01–372469.32)	8002.71 (10072.05–6361.28)	0.01 (−0.05 to 0.08)
Male	112446.49 (140839.67–90663.55)	5181.55 (6507.66–4155.14)	281217.38 (353875.19–225148.66)	5557.22 (7007.93–4432.15)	0.06 (0–0.12)
Socio-demographic index					
High SDI	69821.84 (86029.15–57113.54)	4859.59 (5986.89–3977.34)	135149.64 (171390.92–107333.22)	4984.08 (6311.72–3970.36)	0 (−0.08 to 0.09)
High-middle SDI	85214.08 (106686.15–68353.94)	6845.98 (8582.42–5474.83)	174513.45 (218233.51–138634.61)	6805.33 (8525.27–5391.03)	−0.18 (−0.23 to −0.13)
Middle SDI	69247.1 (86810.65–55655.58)	5815.77 (7316.96–4643.1)	221378.45 (277062.59–177697.13)	6672.44 (8372.84–5333.99)	0.24 (0.18–0.3)
Low-middle SDI	60869.06 (77419.43–47970.89)	8692.22 (11081.52–6807.88)	157225.28 (200280.06–124055.77)	9113.63 (11633.98–7161.16)	−0.14 (−0.26 to −0.03)
Low SDI	27141.73 (35006.25–21035.38)	10724.6 (13900.19–8236.95)	60568.16 (78685.33–46667.44)	10748.45 (13994.39–8232.15)	−0.22 (−0.31 to −0.12)
Region					
Andean Latin America	1352.76 (1776.44–1042.19)	5690.13 (7482.13–4372.26)	4431.35 (5893.58–3311.82)	6134.15 (8163.5–4581.58)	−0.01 (−0.12 to 0.09)
Australasia	1493.47 (1911.34–1172.69)	4852.21 (6216.61–3804.77)	3215.76 (4448.44–2285.83)	4643.89 (6415.88–3304.71)	−0.04 (−0.2 to 0.12)
Caribbean	2573.16 (3385.94–1962.59)	8035.01 (10584.61–6119.44)	5554.2 (7390.63–4156.27)	8275.36 (11002.51–6199.86)	−0.06 (−0.13 to 0.02)
Central Asia	4971.47 (6409.73–3831.97)	8893.51 (11491.06–6840.47)	8352.33 (10925.38–6334.62)	8586.19 (11286.95–6449.75)	−0.3 (−0.37 to −0.23)
Central Europe	12317.9 (15671.1–9675.88)	6333.4 (8070.72–4955.94)	18522.78 (23816.95–14295.51)	6185.75 (7958.83–4773.2)	−0.41 (−0.53 to −0.28)
Central Latin America	6421.09 (8215.77–5055.98)	6712.84 (8604.31–5267.27)	22212.38 (28530.54–17365.88)	7165.88 (9211.02–5593.14)	−0.1 (−0.22 to 0.02)
Central Sub-Saharan Africa	3075.97 (4066.33–2299.58)	12668.91 (16872.4–9368.91)	7421.27 (10112.6–5405.19)	13024.14 (17860.35–9381.3)	0.03 (0–0.07)
East Asia	49484.24 (61611.48–40093.44)	4911.41 (6143.19–3,957)	167633.01 (209055.16–135263.18)	6039.14 (7548.32–4851.07)	0.55 (0.43–0.67)
Eastern Europe	31955.71 (40998.96–24799.41)	8760.61 (11215.11–6813.29)	39994.04 (50791.77–31128.18)	8295.97 (10554.23–6434.99)	−0.58 (−0.7 to−0.45)
Eastern Sub-Saharan Africa	11419.38 (14612.88–8901.18)	13816.81 (17785.72–10670.55)	25641.23 (32907.48–19953.13)	14047.45 (18073.58–10854.37)	−0.11 (−0.17 to −0.04)
High-income Asia Pacific	8200.36 (10170.63–6626.61)	3263.81 (4051.33–2634.9)	20731.5 (26153.49–16489.81)	3491.95 (4396.51–2793.63)	0.41 (0.29–0.52)
High-income North America	18722.85 (23301.87–15152.82)	4067.03 (5060.36–3295.23)	41143.18 (51465.57–33124.33)	4687.26 (5861.4–3774.83)	0.04 (−0.09 to 0.16)
North Africa and Middle East	13443.22 (17575.69–10341.96)	6820.18 (8950.61–5209.03)	38858.46 (51789.55–29314.86)	7265.98 (9704.79–5456.45)	0.11 (0.07–0.15)
Oceania	141.49 (184.99–110.37)	4377.8 (5767.23–3366.4)	356.24 (482.36–264.83)	4443.95 (6035.2–3278.06)	−0.02 (−0.05 to 0.01)
South Asia	60214.14 (76344.56–47707.77)	9225.65 (11723.92–7264.59)	172499.32 (217700.11–137379.27)	9599.31 (12143.58–7610.65)	−0.28 (−0.45 to −0.12)
Southeast Asia	11125.06 (14084.05–8900.15)	3828.46 (4864.88–3039.01)	32839.96 (41615.89–26104.22)	4099.14 (5223.27–3230.19)	0.1 (0.03–0.17)
Southern Latin America	2763.2 (3552.34–2133.76)	4688.02 (6038.59–3611.16)	4992.07 (6690.9–3776.01)	4457.75 (5971.24–3375.31)	−0.41 (−0.51 to −0.31)
Southern Sub-Saharan Africa	3012.58 (3811.17–2381.52)	9647.86 (12238.81–7603.5)	6993.65 (8917.75–5511.01)	10288.81 (13141.32–8073.1)	−0.01 (−0.12 to 0.1)
Tropical Latin America	8673.42 (10839.05–6981.24)	7979.2 (9998.88–6399.04)	26001.93 (32649.87–20686.6)	7999.43 (10053.57–6353.85)	−0.26 (−0.36 to −0.17)
Western Europe	50046.43 (61222.4–40974.37)	6598.52 (8065.78–5412.7)	78169.82 (101396.15–60714.04)	6656.95 (8611.02–5199.9)	0.09 (0–0.18)
Western Sub-Saharan Africa	11252.72 (14396.82–8731.85)	11397.51 (14617.39–8793.43)	23918.44 (30820.36–18365.63)	11463.45 (14809.47–8748.01)	−0.04 (−0.13 to 0.05)

**Figure 2:**
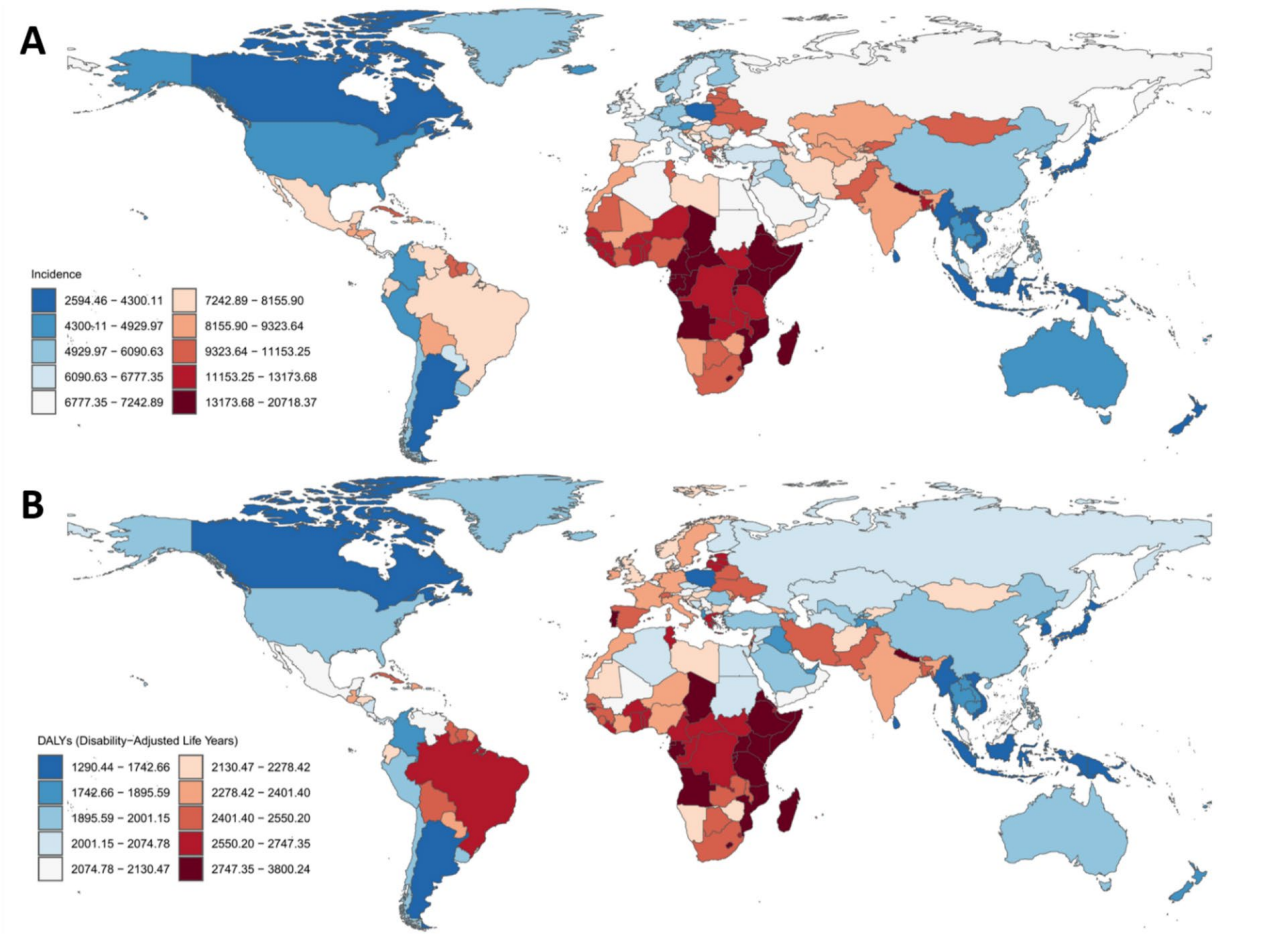
Global distribution of age-standardized incidence and disability-adjusted life years rates for mental disorders among people over 60 years across 204 countries and territories in 2021. **(A)** Age-standardized incidence rate (ASIR). **(B)** Age-standardized DALY rate (ASDR). Green–lowest value; Yellow–highest value.

**Figure 3:**
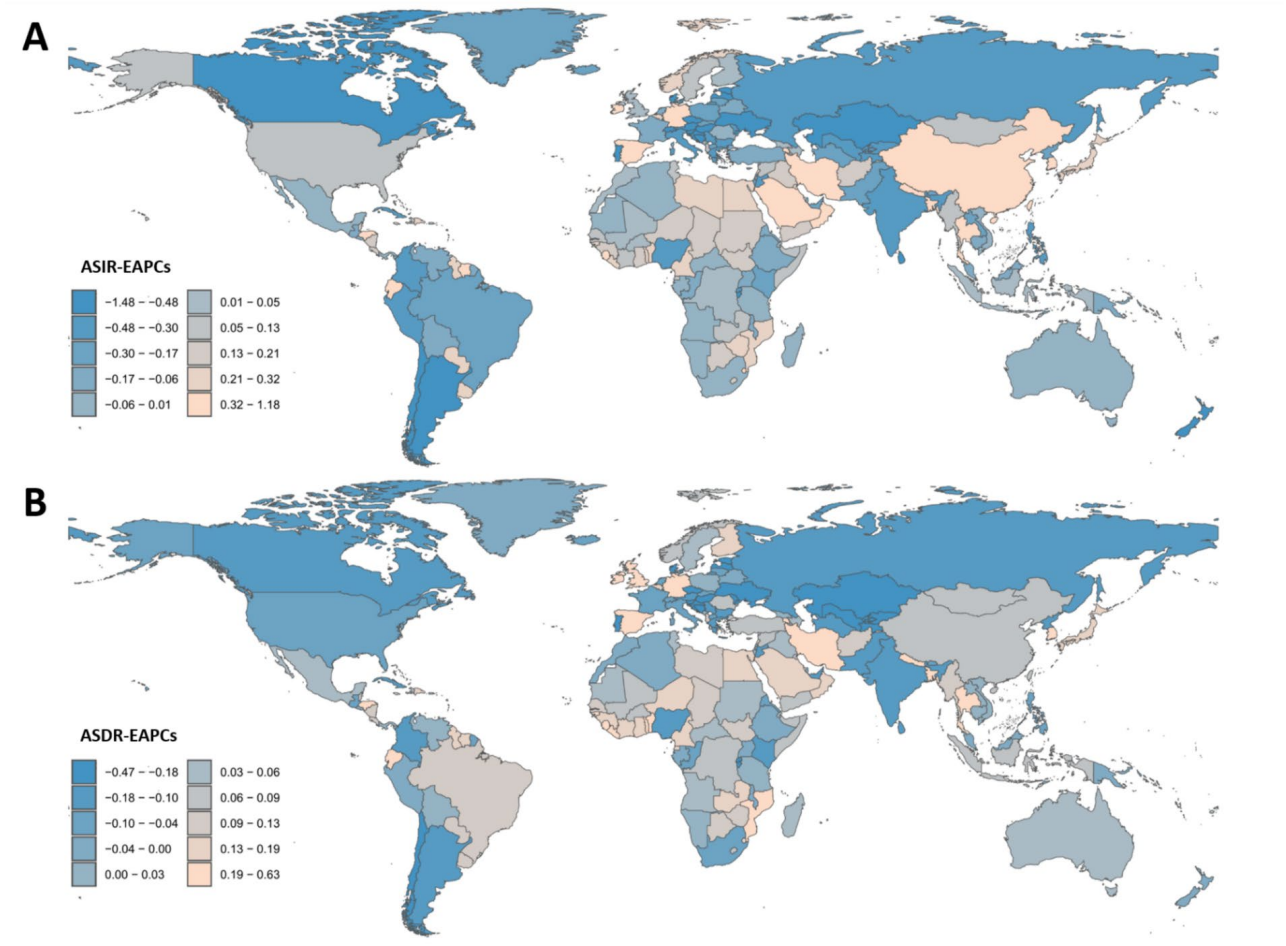
Estimated annual percentage change and age-standardized incidence and disability-adjusted life years rates of mental disorders in the people over 60 years in 204 Countries/territories from 1990 to 2021. The EAPC was calculated for the period 1990–2021. **(A)** EAPC of ASIR; **(B)** EAPC of ASDR.

Regionally, Central Sub-Saharan Africa had the highest ASIR (13,024.14 per 100,000; 95% UI: 9,381.3–17,860.35), nearly three times that of the lowest-ranking region, Oceania (4,643.89; 95% UI: 3,304.71–6,415.88) (Table 1; Figure 2A). The most pronounced decline in ASIR was observed in Eastern Europe (EAPC: −0.58; 95% CI: −0.7 to −0.45), whereas East Asia exhibited the fastest increase (EAPC: 0.55; 95% CI: 0.43–0.67) (Table 1; Figure 3A). When stratified by SDI, low-SDI regions had the highest ASIR (10,748.45; 95% UI: 8,232.15–13,994.39) but also the largest decline (EAPC: −0.22; 95% CI: −0.31 to −0.12). In contrast, middle-high SDI regions had the lowest ASIR (6,805.33; 95% UI: 5,391.03–8,525.27) and showed a moderate decline (EAPC: −0.18; 95% CI: −0.23 to −0.13) (Table 1; Figure 3A). These findings highlight persistent disparities, with low-resource regions bearing the highest burden, while the rising incidence in East Asia may be linked to demographic shifts and disparities in healthcare access.

In 2021, the global age-standardized DALY rate (ASDR) due to mental disorders among individuals aged 60 + years was 2,095.43 per 100,000 population (95% UI: 1,537.49–2,718.99), reflecting a slight increase from 1990 (2,003.53; 95% UI: 1,464.06–2,598.96). However, the EAPC was 0 (95% CI: −0.04 to 0.05), suggesting an overall stable burden (Table 2; Figures 1B, 2B, 3B). Among the 21 GBD regions, Central Sub-Saharan Africa had the highest ASDR (2,681.48; 95% UI: 1,861.61–3,601.57), while the high-income Asia Pacific region had the lowest (1,487.41; 95% UI: 1,113.79–1,869.56). The largest increase in EAPC was observed in the high-income Asia Pacific region (0.20; 95% CI: 0.16–0.23), whereas Eastern Europe experienced the most significant decline (−0.25; 95% CI: −0.31 to −0.18) (Table 2; Figures 2B, 3B). When stratified by SDI, low-SDI regions had the highest ASDR (2,423.86; 95% UI: 1,726.38–3,173.06), while high-SDI regions had the lowest (1,934.16; 95% UI: 1,429.33–2,502.99). The EAPC analysis revealed a notable increase in middle-SDI regions (0.09; 95% CI: 0.03–0.15) and a marked decline in high-middle SDI regions (−0.07; 95% CI: −0.11 to −0.04) (Table 2; Figure 3B). These results underscore significant geographic disparities, with low-SDI regions and aging populations remaining key intervention priorities, while high-income regions may have achieved effective disease burden control through optimized healthcare systems.

**Table 2:** Age-standardized DALY rates and absolute case counts (in hundreds) of mental disorders among people over 60 years from 1990 to 2021.

	1990		2021		EAPC (95%CI)
	Numbers (95%UI)	ASPR (95%UI)	Numbers (95%UI)	ASPR (95%UI)	
Global	98586.12 (127865.95–72058.05)	2003.53 (2598.96–1464.06)	229541.83 (297809.19–168476.73)	2095.43 (2718.99–1537.49)	0 (−0.04 to 0.05)
Sex					
Female	59214.7 (77340.57–42910.7)	2198.26 (2871.1–1593.36)	134934.51 (176320.15–98296.29)	2307.69 (3016.21–1680.61)	−0.02 (−0.07 to 0.04)
Male	39371.42 (50439.89–29259.27)	1760.22 (2255.15–1305.97)	94607.32 (121322.06–70243.37)	1850.44 (2373.5–1373.08)	0.07 (0.04–0.09)
Socio-demographic index					
High SDI	27030.06 (34854.16–19917.52)	1881.82 (2427.13–1386.99)	51720.99 (66884.79–38219.99)	1934.16 (2502.99–1429.33)	−0.01 (−0.06 to 0.05)
High-middle SDI	26068.45 (33910.94–19012.76)	2051.93 (2670.5–1495.77)	53850.37 (69902.34–39525.4)	2092.87 (2717.79–1535.05)	−0.07 (−0.11 to −0.04)
Middle SDI	23,443 (30440.25–17193.06)	1910.48 (2481.74–1399.68)	69546.25 (90434.28–50937.73)	2073.44 (2697.07–1517.53)	0.09 (0.03–0.15)
Low-middle SDI	15733.35 (20388.23–11379.68)	2200.51 (2854–1592.28)	40254.14 (52335.53–29436.62)	2304.31 (2996.81–1685.66)	−0.02 (−0.09 to 0.05)
Low SDI	6198.42 (8078.53–4424.47)	2375.8 (3105.62–1696.51)	13968.96 (18243.22–9957.14)	2423.86 (3173.06–1726.38)	−0.07 (−0.13 to −0.01)
Region					
Andean Latin America	476.44 (630.51–341.17)	1982.57 (2623.01–1420.31)	1544.49 (2038.38–1107.14)	2131.9 (2814.4–1528.13)	0.07 (−0.01 to 0.14)
Australasia	608.8 (787.81–444.74)	1968.34 (2547.72–1437.63)	1341.58 (1749.54–985.06)	1963.9 (2560.97–1439.85)	0.03 (0–0.06)
Caribbean	711.12 (946.37–508.44)	2207.89 (2939.85–1577.77)	1520.09 (2019.83–1104.01)	2268.18 (3013.44–1647.62)	−0.02 (−0.07 to 0.03)
Central Asia	1170.95 (1543.83–845.73)	2054.17 (2705.71–1484.07)	2031.78 (2697.26–1462.01)	2030.62 (2695.85–1458.62)	−0.16 (−0.21 to −0.12)
Central Europe	3746.66 (4897.57–2753.04)	1896.53 (2477.16–1393.5)	5789.92 (7525.03–4236.83)	1943.82 (2527.01–1421.9)	−0.11 (−0.18 to −0.04)
Central Latin America	1882.2 (2448.92–1364.51)	1934.26 (2516.46–1402.04)	6398.96 (8340.2–4587.01)	2051.37 (2673.66–1471.04)	0 (−0.06 to 0.07)
Central Sub-Saharan Africa	657.34 (877.2–459.86)	2593.07 (3472.05–1812.17)	1583.61 (2120.89–1098.28)	2681.48 (3601.57–1861.61)	0.08 (0.05–0.1)
East Asia	19113.5 (24799.38–14161.54)	1819.7 (2363.75–1345.39)	55699.37 (72311.96–41281.9)	1988.73 (2584.05–1472.04)	0.08 (0.02–0.15)
Eastern Europe	8011.66 (10459.77–5719.87)	2165.83 (2829.3–1546.71)	10483.61 (13624.62–7588.5)	2162.86 (2811.42–1563.33)	−0.25 (−0.31 to −0.18)
Eastern Sub-Saharan Africa	2362.26 (3100.54–1668.94)	2777.3 (3657.01–1961.04)	5346.46 (7027.67–3797.21)	2859.97 (3768.02–2029.53)	−0.01 (−0.05 to 0.03)
High-income Asia Pacific	3607.6 (4547.87–2702.25)	1407.87 (1775.71–1053.98)	8350.15 (10500.19–6248.5)	1487.41 (1869.56–1113.79)	0.2 (0.16–0.23)
High-income North America	8360.91 (10699.03–6208.52)	1827.55 (2339.2–1357.4)	16844.48 (21561.2–12590.65)	1925.05 (2464.25–1438.61)	−0.1 (−0.2 to 0)
North Africa and Middle East	4092.35 (5371.52–2954.22)	2036.06 (2672.1–1469.27)	11485.08 (15177.09–8213.51)	2127.47 (2807.52–1523.48)	0.1 (0.07–0.13)
Oceania	57.01 (75.02–41.44)	1697.67 (2232.36–1233.27)	142.52 (190.94–103.02)	1725.78 (2306.33–1247.95)	0 (−0.01 to 0.02)
South Asia	15007.82 (19357.69–10890.12)	2250.28 (2904.16–1633.7)	42597.94 (55264.56–31061.16)	2343.02 (3041.02–1709.29)	−0.11 (−0.21 to 0)
Southeast Asia	4792.85 (6222.56–3521.39)	1606.03 (2083.54–1178.84)	13884.34 (18187.66–10209.72)	1699.11 (2222.23–1249.29)	0.1 (0.07–0.14)
Southern Latin America	1076.52 (1396.94–789.21)	1805.41 (2343.89–1322.85)	2036.48 (2668.69–1465.56)	1826.42 (2393.89–1313.86)	−0.11 (−0.17 to −0.05)
Southern Sub-Saharan Africa	732.5 (959.43–530.94)	2291.83 (3004.48–1660.37)	1670.22 (2178.38–1213.98)	2399.6 (3132.52–1743.4)	−0.01 (−0.08 to 0.06)
Tropical Latin America	2652.32 (3467.11–1924.12)	2406.36 (3143.26–1747.36)	8512.16 (11117.34–6120.01)	2608.77 (3406.09–1876.13)	0.13 (0–0.25)
Western Europe	17060.23 (22223.94–12427.74)	2258.8 (2943.85–1645.92)	27080.96 (35417.72–19717.49)	2356.36 (3083.35–1714.38)	0.14 (0.08–0.2)
Western Sub-Saharan Africa	2405.06 (3146.55–1707.88)	2370.04 (3110.38–1681.83)	5197.62 (6806.22–3685.26)	2413.64 (3169.45–1709.5)	0.03 (−0.01 to 0.08)

Across all age groups, the burden of mental disorders was consistently higher in females than males. The ASIR was 7,483.7 per 100,000 (95% UI: 6,004.6–9,367.12) in females compared to 5,181.55 (95% UI: 4,155.14–6,507.66) in males (Table 1). Similarly, the ASDR was 2,198.26 (95%UI: 1,593.36–2,871.1) in females versus 1,760.22 (95% UI: 1,305.97–2,255.15) in males (Table 2). While incidence trends remained stable for both sexes (female EAPC: 0.01; 95% CI: −0.05 to 0.08; male EAPC: 0.06; 95% CI: 0–0.12) (Table 1), DALY rates exhibited divergent patterns: females showed a slight decline (−0.02; 95%CI: −0.07 to 0.04), whereas males experienced a significant increase (0.07; 95%CI: 0.04–0.09) (Table 2). These findings highlight the urgent need for sex-specific interventions, particularly to address the growing disease burden among aging males, while continuing to manage the persistently higher absolute burden in females.

### 3.2 Association analysis with SDI

**Figure 4:**
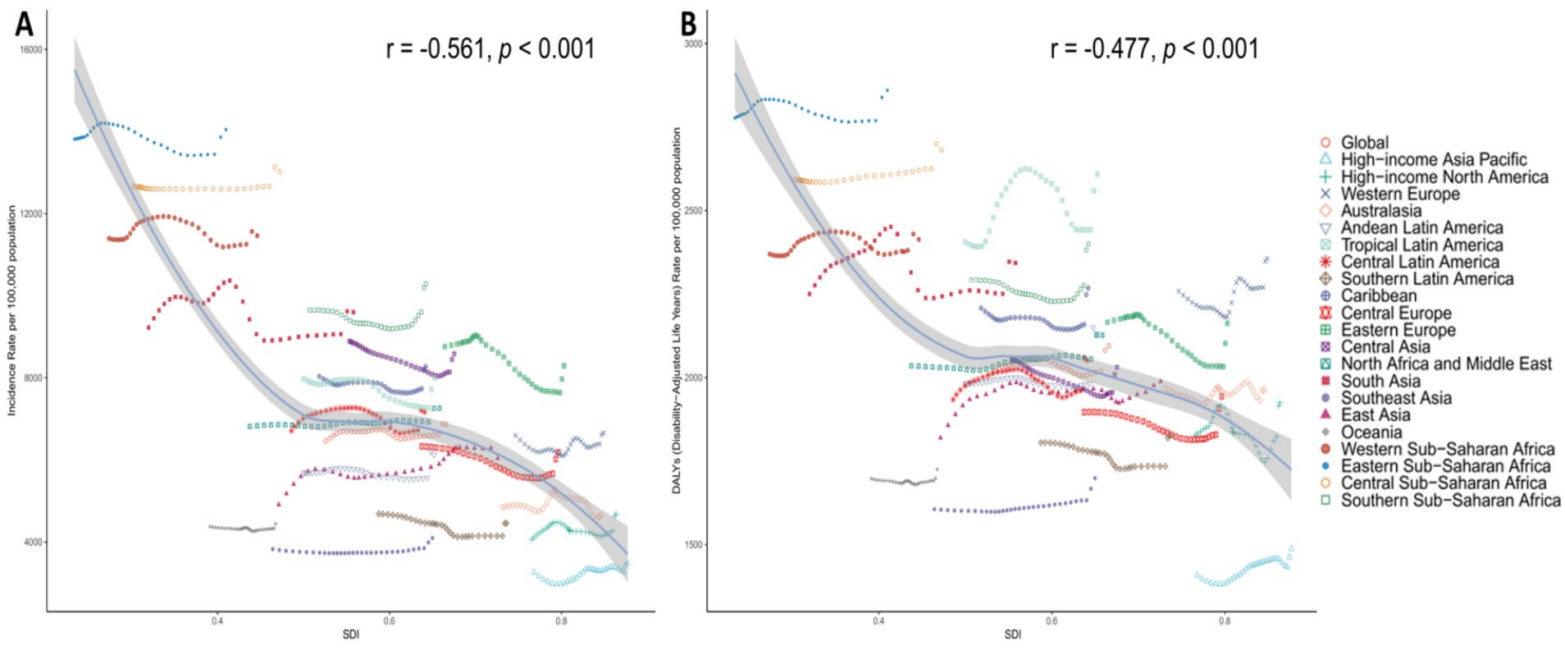
Age-standardized rates of mental disorders among people over 60 years in Relation to SDI in 2021 Among 21 GBD Regions. **(A)** Age-standardized incidence rate (ASIR). **(B)** Age-standardized DALY rate (ASDR).

Figure 4 and Supplementary Figure S1 illustrate the relationship between the ASIR and ASDR of mental disorders among the people over 60 years, and the SDI globally, regionally, and nationally in 2021. Figure 4A shows a significant negative correlation between ASIR and SDI (*r* = −0.561, *p* < 0.001), indicating that regions with lower SDI have higher ASIR. Figure 4B shows a significant negative correlation between ASDR and SDI (*r* = −0.477, *p* < 0.001), similarly indicating that regions with lower SDI have higher ASDR. Supplementary Figure S1 further breaks down the analysis to the national level. Supplementary Figure S1A shows a significant negative correlation between ASIR and SDI (*r* = −0.522, *p* < 0.001), and Supplementary Figure S1B shows a significant negative correlation between ASDR and SDI (*r* = −0.335, *p* < 0.001). These results suggest that the burden of mental disorders is more severe in regions with lower SDI, highlighting the need to develop more targeted interventions for these areas.

Supplementary Figure S2 presents the relationship between the ASIR and ASDR of mental disorders among the people over 60 years in 2021, and the SDI as well as the EAPC across 204 countries globally. Supplementary Figure S2A shows a weak relationship between incidence and EAPC, with a correlation coefficient of 0.11 and a *p*-value of 0.11. Supplementary Figure S2B reveals a significant positive correlation between SDI and EAPC, with a correlation coefficient of 0.21 and a *p*-value of 0.0027, indicating that regions with higher SDI have higher EAPC. Supplementary Figure S2C shows a similarly weak relationship between DALYs and EAPC, with a correlation coefficient of 0.11 and a *p*-value of 0.11. Supplementary Figure S2D shows a significant positive correlation between SDI and the EAPC of DALYs, with a correlation coefficient of 0.24 and a *p*-value of 0.00064. These results suggest that regions with higher SDI exhibit more significant changes in the incidence of mental disorders and DALYs, indicating that the level of socio-economic development may have an important impact on the epidemiological trends of mental disorders.

### 3.3 Health inequality analysis

**Figure 5:**
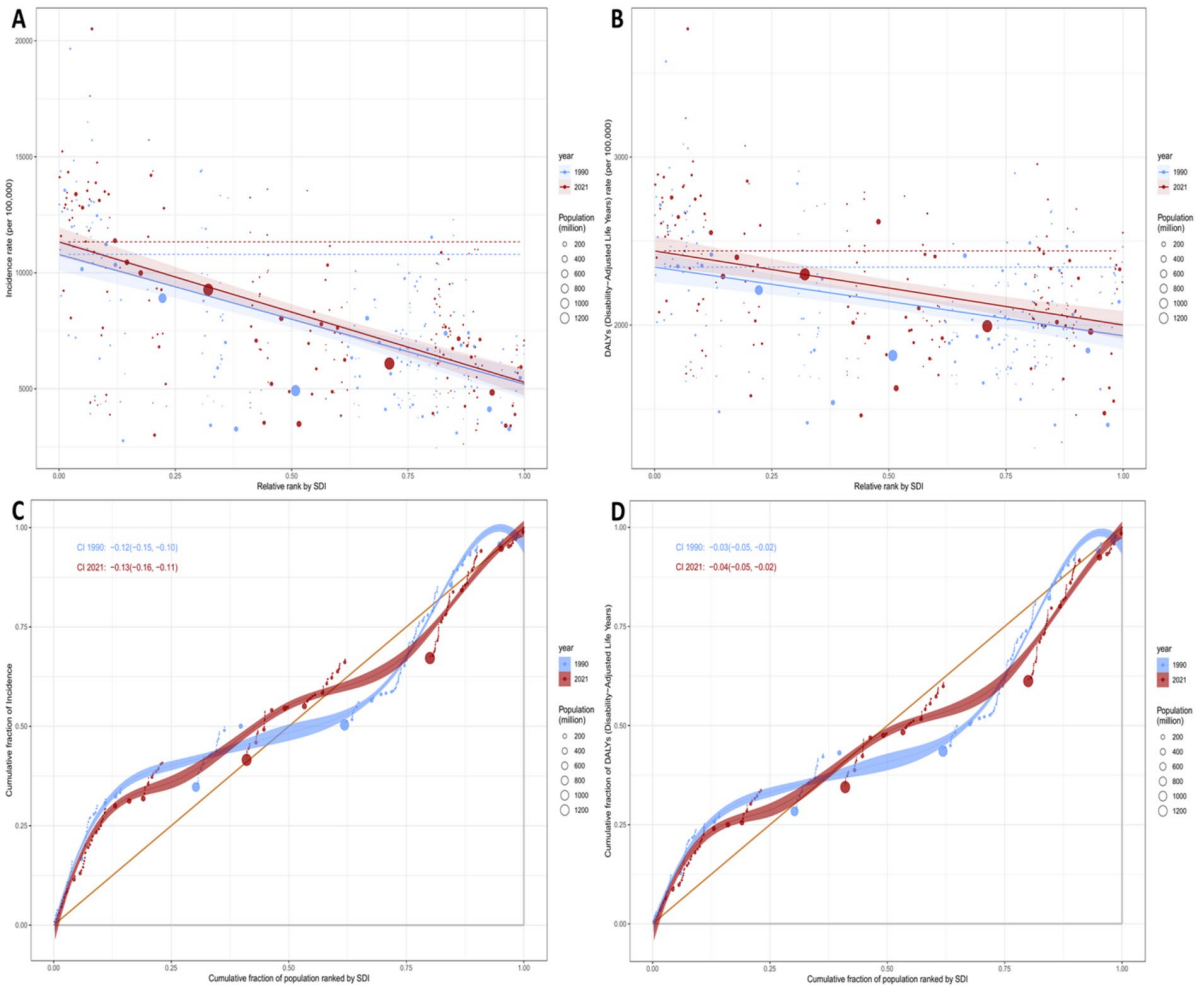
Regression curves and concentration curves for health inequality in the incidence and DALYs of mental disorders among people over 60 years globally in 1990 and 2021. **(A,B)** The slope indices of inequality, depicting the relationship between SDI and age-standardized rates, with points representing individual countries and regions weighted by population size. **(C,D)** Present concentration indices, which quantify relative inequality by integrating the area under the Lorenz curves, aligning the distribution of incidence and DALYs with the population distribution stratified by SDI. Blue represents data from 1990, and red represents data from 2021.

Figure 5 and Supplementary Table S1, provides a detailed analysis of the absolute and relative health inequalities in the incidence and DALYs of mental disorders among the people over 60 years globally in 1990 and 2021. Figures 5A,B show the relationship between the age-standardized rates of incidence and DALYs and the SDI, revealing health inequalities across different SDI levels through regression curves. Figures 5C,D further quantify health inequalities by displaying the cumulative burden distribution across different SDI levels using Lorenz curves for cumulative incidence and DALYs. The slope coefficients in Supplementary Table S1 reflect the trends in health inequalities. For incidence, the slope coefficient was −5587.34 in 1990 and −6047.42 in 2021, indicating that over time, the burden of incidence in low-SDI regions has relatively increased, with a worsening of absolute inequality. For DALYs, the slope coefficient was −408.64 in 1990 and −440.72 in 2021, showing an increase in the burden of DALYs in low-SDI regions, albeit with a relatively smaller magnitude of change. Relative inequalities were measured using Lorenz curves and concentration indices. The Lorenz curves in Figures 5C,D illustrate the distribution of cumulative incidence and DALYs across different SDI levels in 1990 and 2021. The 95% confidence interval (CI) for the cumulative incidence Lorenz curve in 1990 was −0.12 to −0.10, while in 2021, it was −0.13 to −0.11, indicating a higher burden of cumulative incidence in low-SDI regions, with this relative inequality persisting over time. Similarly, the 95% CI for the cumulative distribution of DALYs in 1990 was−0.03 to −0.02, and in 2021, it was −0.04 to −0.02, showing a higher burden of DALYs in low-SDI regions. By comparing the Lorenz curves and their confidence intervals between 1990 and 2021, it is evident that the burden in low-SDI regions has increased, further confirming the exacerbation of relative inequalities. These findings underscore the need for more targeted interventions in low-SDI regions to reduce health inequalities.

### 3.4 Frontier analysis

**Figure 6:**
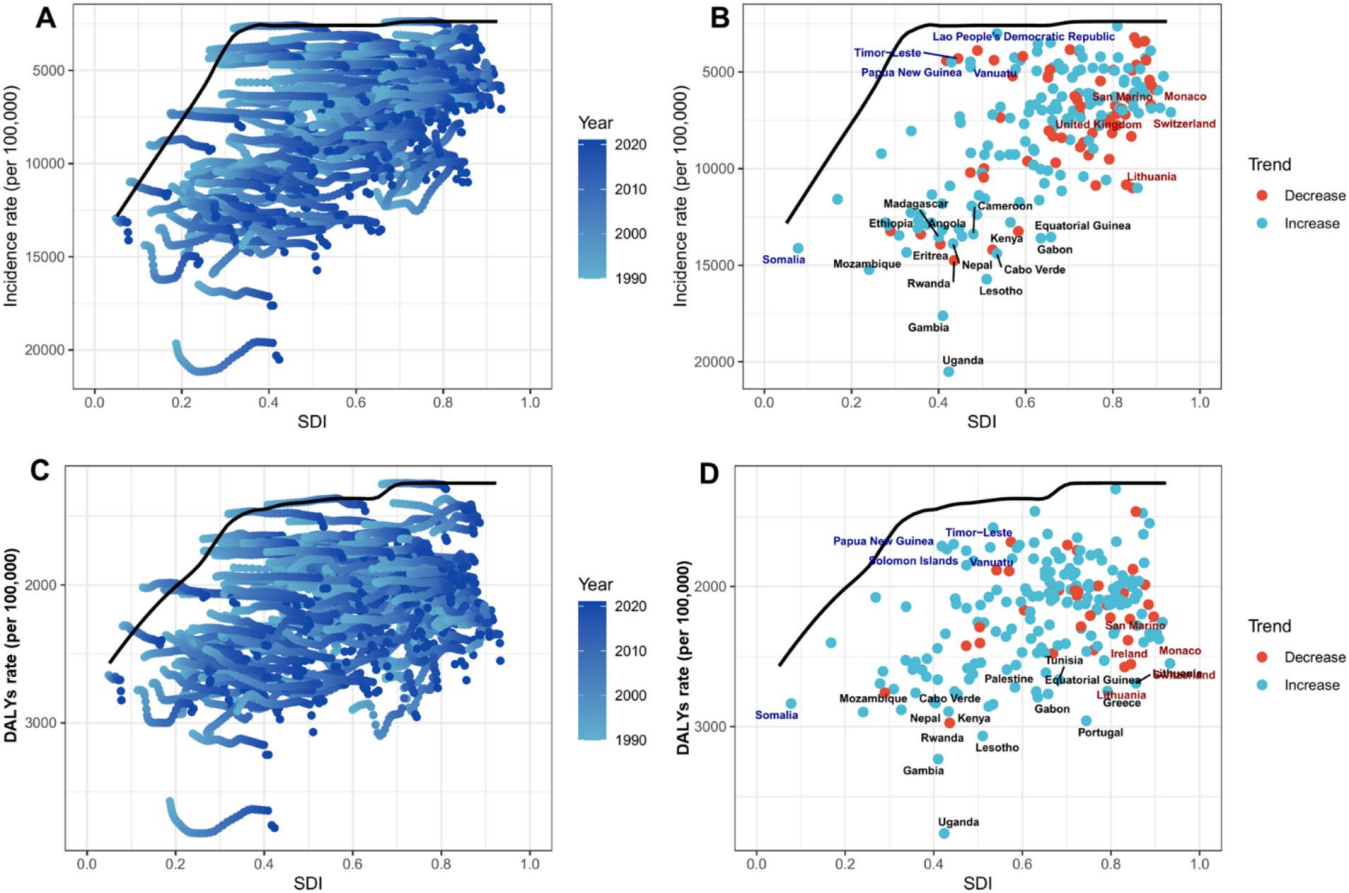
Frontier analysis of the relationship between SDI and ASR of mental disorders among people over 60 years in 204 countries and regions. In **(A,C)**, the color gradient from light green (1990) to dark green (2021) represents the changeover years. In **(B,D)**, each point represents a specific country or region in 2021, with the frontier line shown in black. Blue indicates low SDI with the smallest difference from the frontier, while red indicates high SDI with the largest difference from the frontier. The direction of change in ASR from 1990 to 2021 is indicated by the color of the points, with orange representing a decline and green representing an increase. **(A,B)** The age-standardized incidence rate (ASIR), while **(C,D)** the age-standardized DALY rate (ASDR). The frontier line represents the lowest age-standardized rate (ASIR or ASDR) observed in 2021 at each given level of the SDI; it denotes the theoretically attainable minimum disease burden under current development conditions.

Figure 6 presents a frontier analysis of the relationship between the SDI and the age-standardized rate (ASR) of mental disorders among the people over 60 years in 204 countries and regions from 1990 to 2021. Figures 6A,C show the temporal trends of the ASIR and ASDR, respectively. The color gradient ranging from light green (1990) to dark green (2021) illustrates that as SDI improves, the disease burden tends to increase. The frontier plots (Figures 6B,D) reveal significant differences among countries in 2021. Low-SDI countries (blue), such as Timor-Leste and Papua New Guinea, have incidence rates close to 15,000 per 100,000 population, closely aligning with the epidemiological frontier. In contrast, high-SDI countries (red), such as Switzerland and Monaco, show a substantial gap between the observed incidence rates and the theoretically achievable incidence rates. Notably, 32% of countries exhibit an upward trend in ASIR (indicated by green dots), particularly in East Asia and Oceania, while a downward trend (indicated by orange dots) is concentrated in Eastern Europe (e.g., Lithuania) and Southern Africa. The DALY frontier (Figure 6D) shows a similar divergence, with ASDR in low-SDI countries being 2.3 times higher than in high-SDI regions. Despite an overall increase in global SDI, persistent inequalities remain in the access to mental health care for the older adults.

### 3.5 National-level analysis

According to the 2021 Global Burden of Disease (GBD) data, the ASIR of mental disorders among people over 60 years shows significant variation across 204 countries globally. Uganda has the highest ASIR, at 20,513.24 per 100,000 population [95% uncertainty interval (UI): 14,496–28,497], while Singapore has the lowest ASIR, at 3,492.62 per 100,000 population (95% UI: 2,485.34–4,883.32) (Supplementary Table S3; Figure 2A). From 1990 to 2021, the EAPC in ASIR ranges from −1.47 in Singapore (95% confidence interval [CI]: −1.66 to −1.27), indicating the most significant decline, to 1.17 in Spain (95% CI: 0.89–1.45), reflecting the most significant increase (Supplementary Table S3; Figure 3A). Notably, high-income regions such as Singapore and Western Europe show declining trends, while low- and middle-income regions (e.g., sub-Saharan Africa and Latin America) exhibit stable or rising ASIR. This may be related to differences in healthcare access, diagnostic capacity, and the dynamics of the older adults. These findings highlight the need for targeted interventions to address the growing burden of mental disorders among the older adults, especially in resource-limited settings.

Similarly, the age-standardized disability-adjusted life year rate (ASDR) of mental disorders among people over 60 years also shows significant variation across the 204 countries. Uganda has the highest ASDR, at 3,762.62 per 100,000 population (95% UI: 2,519.99–5,271.42), while Brunei Darussalam has the lowest ASDR, at 1,303.47 per 100,000 population (95% UI: 958.88–1,673.47) (Supplementary Table S4; Figure 2B). From 1990 to 2021, the EAPC in ASDR ranges from −0.47 in Singapore (95% CI: −0.54 to −0.40), indicating the most significant decline, to 0.42 in South Korea (95% CI: 0.30–0.53), reflecting the most significant increase (Supplementary Table S3; Figure 3B). Notably, high-income regions such as Singapore and Western Europe (e.g., Slovenia, EAPC: −0.39) show a significant reduction in ASDR, while lower-middle-income countries, particularly in sub-Saharan Africa (e.g., Sierra Leone, EAPC: 0.28) and Southeast Asia, face an increasing burden. These differences highlight varying trends influenced by healthcare access, the older adults, and diagnostic practices, emphasizing the need for targeted mental health interventions in high-burden regions.

### 3.6 Future trend projections

**Figure 7:**
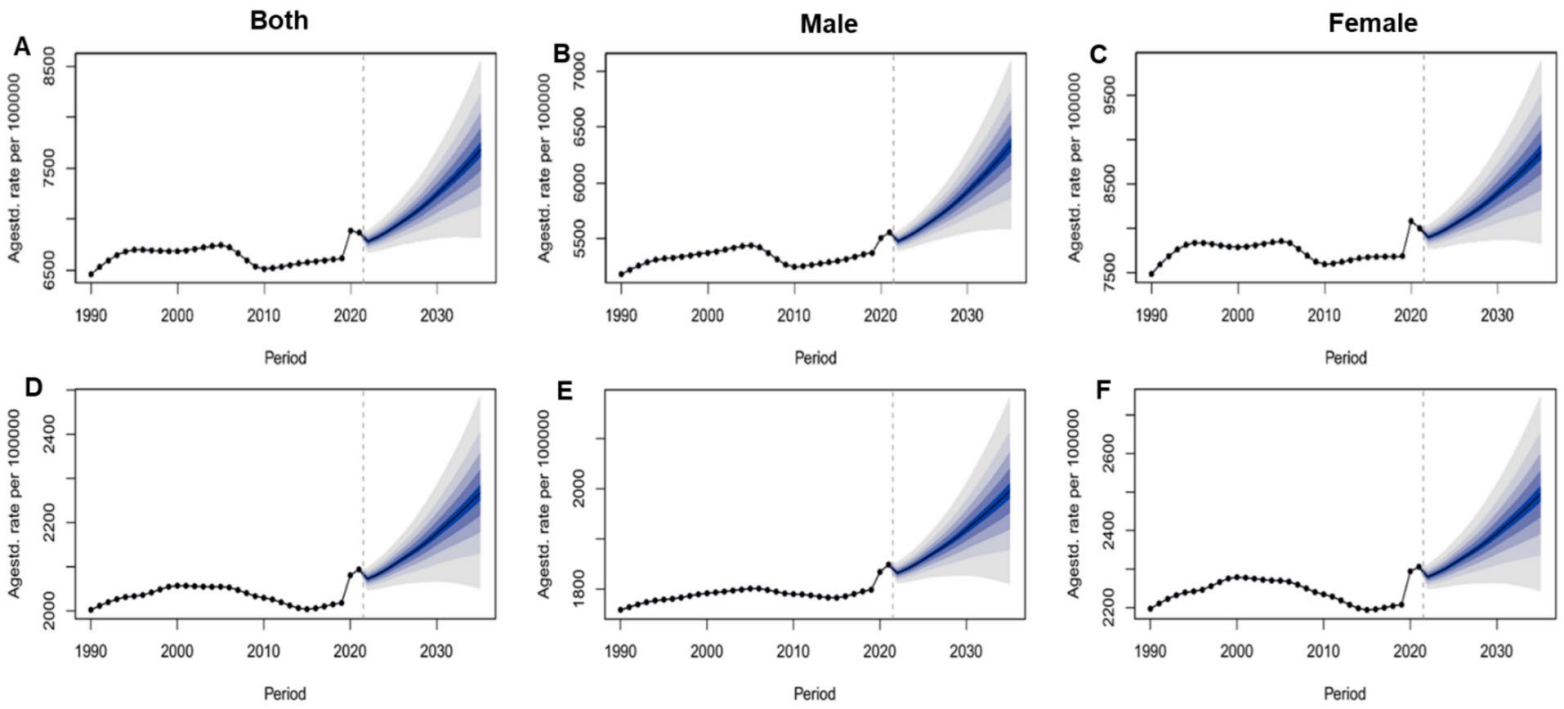
Global projections of age-standardized incidence rate and age-standardized disability-adjusted life year rate of mental disorders among people over 60 years for males, females, and both sexes, from 1990 to 2035. **(A)** ASIR of Both sex; **(B)** ASIR of Male; **(C)** ASIR of Female; **(D)** ASDR of Both sex; **(E)** ASDR of Male; **(F)** ASDR of Female.

Based on data from the 2021 Global Burden of Disease (GBD) study and projections using the Bayesian Age-Period-Cohort (BAPC) model (Supplementary Table S2; Figure 7), the burden of mental health disorders among the global people over 60 years from 1990 to 2035 shows significant gender differences and concerning epidemiological trends. Figure 7 illustrates the projected trends of ASIR and age-standardized disability-adjusted life year rate (ASDR) of mental disorders among people over 60 years globally. From 1990 to 2035, both the combined ASIR and ASDR for both sexes are projected to increase continuously. Notably, females have significantly higher ASIR and ASDR than males across all time periods, with a more pronounced upward trend since 2010 (Figures 7C,F).

Supplementary Table S2 provides detailed year-by-year projections of ASIR and ASDR for mental disorders among the older adults from 2021 to 2035. The ASIR is projected to increase from 6,866.46 per 100,000 population in 2021 to 7,679.28 per 100,000 population in 2035. The ASDR is projected to rise from 2,093.83 per 100,000 population in 2021 to 2,494.53 per 100,000 population in 2035. For females, the ASIR is projected to increase from 8,000.19 per 100,000 population in 2021 to 8,856.09 per 100,000 population in 2035, while the ASDR is projected to rise from 2,305.82 per 100,000 population in 2021 to 2,494.53 per 100,000 population in 2035, both of which are higher than the corresponding values for males. These results highlight the increasing burden of mental health disorders with global population aging, particularly the more severe challenges faced by the female population. There is an urgent need for targeted public health interventions to address this challenge.

Supplementary Figure S4 projects that from 2021 to 2036, the ASIR of anxiety disorders will stabilize initially before rising, while depressive disorders show a continued upward trend. For bipolar disorder, ASIR is expected to increase moderately, whereas schizophrenia exhibits a slight decline. In terms of disability burden (ASDR), anxiety disorders and bipolar disorder demonstrate stable or marginally decreasing trends, while depressive disorders and schizophrenia show modest reductions, with uncertainty intervals narrowing in later years. These projections suggest divergent epidemiological patterns across mental disorders in older adults (≥60 years).

### 3.7 Subtype analysis of mental disorders

Supplementary Figure S3A shows that depressive disorders (blue section) have the highest proportion across all regions and SDI strata, especially in developed areas (such as high SDI and Western Europe) where the proportion exceeds 40%; anxiety disorders (orange section) are the second most common mental illness, with a particularly prominent proportion in high-income Asia-Pacific regions (about 35%). There are significant regional differences in the distribution of mental illness types: high SDI areas are mainly characterized by depression and anxiety disorders, while low SDI areas such as Sub-Saharan Africa have significantly higher proportions of schizophrenia (purple section) and bipolar disorder (green section). Supplementary Figure S3B shows that Anxiety disorders (red section) have the highest proportion of DALY rates among people over 60, especially in developed areas (such as high SDI, Western Europe, and high-income North America) where they generally exceed 30%, becoming the primary factor in the global burden of mental health among the older adults. Depressive disorders (dark blue section) follow closely behind, forming the main burden together with anxiety disorders, with the combined proportion of the two exceeding 50% in most regions, while the proportion of schizophrenia (purple section) is relatively higher in low-income areas (such as Sub-Saharan Africa) (about 10–15%).

## 4 Discussion

This global burden of disease study reveals persistent and widening disparities in mental disorders among people over 60 years, with low-resource regions bearing the highest burden while facing the greatest healthcare access barriers. The analysis demonstrates a clear socioeconomic gradient, where lower SDI regions exhibit disproportionately higher incidence and disability rates, particularly for severe mental illnesses like schizophrenia, whereas high-SDI areas show predominance of mood disorders. Notably, the burden shows concerning gender disparities, with women consistently experiencing higher rates but men demonstrating faster-growing disability trends. The projected increase in global burden, especially in rapidly aging populations, underscores the urgent need for developmentally appropriate interventions—integrating community-based mental healthcare in low-SDI regions while addressing emerging challenges in high-SDI settings through prevention-focused strategies. These findings highlight how current mental health systems fail to achieve equitable gains with socioeconomic development, calling for fundamentally reoriented approaches that prioritize both immediate service gaps and long-term structural determinants of mental health in aging populations worldwide.

Global Burden of Disease (GBD) 2021 findings delineate the contemporary epidemiology of mental disorders among older adults (≥60 years), underscoring persistent geographic and sex-specific disparities. Although the global ASIR remained stable between 1990 and 2021, the burden is disproportionately concentrated in resource-limited settings. Central Sub-Saharan Africa exhibited the highest ASIR and ASDR, aligning with the inverse care law—populations with the greatest need often have the least access to mental-health services (20). Notably, the steepest ASIR increase was observed in East Asia, likely reflecting rapid population aging concomitant with fragmented health systems (21). This divergence is further driven by East Asia–specific sociodemographic factors: (1) accelerated aging—Japan and South Korea lead globally in the pace of demographic transition, while the incidence of mood disorders and dementias rises exponentially with age (22); (2) differential healthcare access—despite high economic development in parts of the region, coverage of mental-health services for older adults remains inadequate, and stigma-related underutilization persists (23); (3) urbanization and psychosocial stress—nuclearization of family structures (increasing numbers of “empty-nest” older adults) and intense social competition may heighten psychological distress (24); and (4) improved case ascertainment—recent scale-up of mental-health screening programs has identified previously undiagnosed cases. Future longitudinal analyses that integrate culturally specific determinants—such as the erosion of filial piety and its impact on family support—together with rigorous policy evaluation are required to optimize regional prevention and control strategies. Conversely, Eastern Europe recorded the largest ASIR decline, suggesting successful public-health interventions (25). Marked sex differences are evident: women bear a higher absolute burden, yet men experienced a steeper increase in DALY rates, highlighting the interplay between biological vulnerability and gendered patterns of healthcare utilization (26). These findings call for targeted policies: (1) expanding community-based mental-health services in low-SDI settings; (2) integrating geriatric mental-health care into primary-health-care systems in rapidly aging societies; and (3) developing sex-specific interventions to address the rising burden among men. The stable global age-standardized death rate (ASDR) (EAPC = 0) masks substantial within-country inequalities, underscoring the imperative to leverage GBD subnational data for equity-oriented resource allocation.

The observed negative correlation between the SDI and ASIRs or ASDRs at the regional level—along with consistent national-level patterns—demonstrates a distinct socioeconomic gradient in the burden of mental disorders among older adults. These findings align with the “dual burden” hypothesis in global mental health, where resource-limited settings face heightened challenges due to higher baseline incidence and constrained healthcare capacity (27, 28). Notably, the correlation was stronger for ASIR than ASDR, suggesting that while low-SDI regions experience more disease onset, their disability burden may be partially offset by shorter life expectancy or competing mortality risks—a phenomenon warranting further investigation through cause-specific DALY decomposition. The positive correlation between SDI and both incidence (*r* = 0.21) and DALY rates (*r* = 0.24) reveals an emerging epidemiological transition: although high-SDI regions currently exhibit lower absolute rates, their mental health outcomes are deteriorating faster, likely driven by aging populations, expanded diagnostic boundaries, or stress-related comorbidities in industrialized societies (29, 30). This dual pattern underscores the need for differentiated strategies—scaling up essential mental health services in low-SDI settings while addressing prevention gaps in high-SDI contexts through integrated care models. The weak correlation between incidence/DALY rates and EAPCs(*r* = 0.11) indicates that temporal trends depend more on contextual factors (e.g., healthcare investments) than baseline burden, highlighting SDI’s role as a predictor of both cross-sectional disparities and longitudinal trajectories in global mental health epidemiology (31, 32).

Our analysis reveals persistent and widening health inequalities in mental disorders among older adults, demonstrating distinct socioeconomic gradients in both absolute and relative metrics. Increasing slope indices for incidence and DALY rates indicate progressive concentration of disease burden in low-SDI regions, with Lorenz curve analyses confirming worsening relative inequality. Frontier analysis further highlights this disparity, showing low-SDI countries (e.g., Timor-Leste) operating at the epidemiological frontier with ASIRs approaching 15,000 per 100,000 –2.3-fold higher than high-SDI nations - suggesting these regions bear the maximum biologically plausible burden under resource constraints. Despite SDI improvements, paradoxical ASIR increases in 32% of countries (particularly East Asia) reflect an emerging “epidemiological transition penalty,” where development brings longer life expectancy coupled with increased mental health vulnerability (33). These findings underscore how current global mental health systems fail to achieve proportional gains with socioeconomic development, as evidenced by widening frontier gaps in high-SDI countries (e.g., Switzerland) (34, 35). The results demand a dual approach: (1) structural interventions addressing basic healthcare access inequities through task-shifted care models in low-SDI regions, and (2) development-sensitive prevention strategies targeting modifiable risk factors (e.g., social isolation, metabolic disorders) in rapidly aging middle-SDI populations. Persistent health inequalities despite three decades of SDI growth highlight the imperative to explicitly incorporate health equity metrics into Sustainable Development Goal frameworks.

Subtype analysis of mental disorders reveals pronounced socioeconomic gradients in their global distribution among older adults. While depressive and anxiety disorders dominate the disease burden in high-SDI regions (collectively >50%), low-SDI areas show proportionally greater burden from schizophrenia and bipolar disorders—likely reflecting both true epidemiological differences and diagnostic patterns. The exceptionally high prevalence of anxiety disorders (35%) in high-income Asia Pacific may relate to cultural factors in symptom reporting, while elevated schizophrenia proportions (10–15%) in sub-Saharan Africa may indicate unmet treatment needs for psychosis (36). These findings suggest mental health interventions must be regionally tailored, prioritizing mood disorders in high-SDI settings while requiring integrated approaches addressing both severe mental illness and depression/anxiety in low-SDI regions. Decades-stable DALY patterns imply current prevention strategies remain inadequate to alter these fundamental distribution patterns. The burden of schizophrenia among older adults in low-SDI settings is disproportionately high and is mediated by several inter-related factors. First, scarce healthcare resources lead to insufficient early intervention, increasing the risk of chronicity. Second, environmental stressors prevalent in low-SDI contexts—such as poverty, malnutrition, and high infectious disease rates—may aggravate neurodevelopmental vulnerabilities (37). Third, weak social-protection systems expose patients to unemployment, loss of family support, and other psychosocial stressors that accelerate disease progression (38). Moreover, cultural stigma and the predominance of traditional healing practices delay or preclude access to evidence-based psychiatric care (39). These structural disadvantages create a vicious cycle in which patients in low-SDI regions are more likely to develop severe illness and experience higher rates of disability. In low-SDI settings, priority should be given to task-shifting models that train community health workers to screen for and deliver basic psychological interventions for depression and anxiety, supported by mobile-health supervision to minimize costs. Sex-specific programs are essential: women benefit from strengthened community support networks and caregiver training to reduce stigma, while men require anonymous hotlines, peer groups, and integration of mental-health services with existing chronic-disease management to address their rapidly rising DALY rates. The regional disparities in disorder subtypes have critical implications for global mental health policy. The concentration of schizophrenia in low-SDI regions mirrors mhGAP’s focus on severe mental illness in resource-limited settings, where <10% receive treatment. Meanwhile, SDG 3.4’s prevention targets are particularly relevant to high-SDI regions, where anxiety/depression predominance suggests unmet needs in aging populations. Our frontier analysis further quantifies the service gaps that mhGAP aims to address—for example, the 2.3-fold higher DALYs in low-SDI regions versus high-SDI areas matches mhGAP’s priority countries.

Several limitations should be considered when interpreting our findings. First, while the GBD 2021 study provides comprehensive estimates through advanced modeling techniques, these estimates primarily reflect national-level patterns and may not fully capture subnational variations in disease burden, particularly in large or heterogeneous countries. Second, data quality and availability vary significantly across regions, with LMICs facing greater challenges in underreporting and diagnostic inconsistencies—issues further compounded by cultural differences in symptom interpretation and help-seeking behaviors that may systematically bias estimates (e.g., somatic presentations of depression in some cultures being misclassified). Third, our reliance on modeled estimates introduces uncertainty for conditions like schizophrenia and bipolar disorders, where resource-limited settings often lack specialized diagnostic capacity. Fourth, while SDI is a robust composite indicator, it cannot fully incorporate contextual factors like cultural stigma or decentralized healthcare systems that directly shape mental health outcomes. Fifth, frontier analysis assumes SDI-defined theoretical minimums without accounting for unmeasured biological or localized environmental risks. Sixth, BAPC projections based on historical trends may not adapt to abrupt disruptions like pandemics or policy reforms. Finally, broad diagnostic categories may obscure clinically important subtypes with distinct epidemiological patterns. These limitations highlight the critical need for: (1) enhanced subnational data collection in LMICs, (2) culturally adapted diagnostic instruments, and (3) modeling frameworks that better integrate sociocultural determinants of mental health.

## 5 Conclusion

This study highlights persistent disparities in mental disorders among older adults, with low-SDI regions facing higher burdens of severe mental illnesses and women experiencing greater absolute impacts. While high-SDI regions show better control of certain disorders, rising trends reveal gaps in current approaches. Our findings call for targeted strategies: expanding community-based care in underserved areas, implementing sex-specific interventions, and addressing social determinants of mental health. These measures are essential for achieving equitable healthy aging in line with global development goals.

## Data Availability

The original contributions presented in the study are included in the article/Supplementary material, further inquiries can be directed to the corresponding author.

## Footnotes

^1^ https://vizhub.healthdata.org/gbd-results/
