# Supplementary material for: Global trends and health inequalities in mental disorders among older adults: a comprehensive analysis using global burden of disease 2021 data
Source: Front Public Health. 2025 Aug 26;13:1644610. doi: 10.3389/fpubh.2025.1644610 (PMC12419478; doi:10.3389/fpubh.2025.1644610)
Supplement: Supplementary file 1 [file Table_1.docx]

**MATERIAL**

**Supplement to “Global Trends and Health Inequalities in Mental Disorders Among Older Adults: A Comprehensive Analysis Using Global Burden of Disease 2021 Data”**

MATERIAL 1

Fig S1 2

Fig S2 3

Fig S3 3

Fig S4 4

Table S1 5

Table S2 5

Table S3 6

Table S4 14


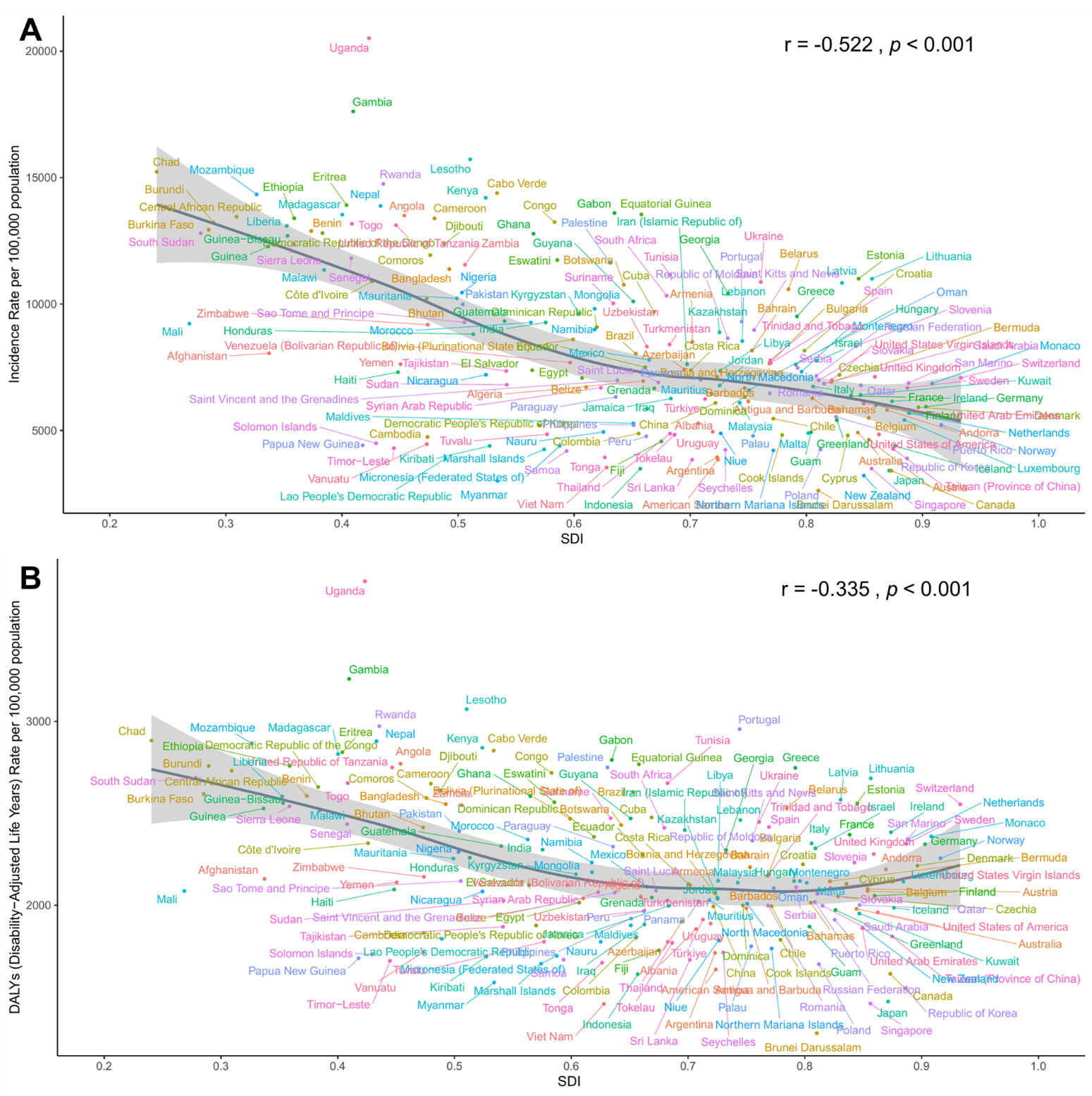


Figure S1: Age-standardized incidence rates (ASIRs) and disability-adjusted life year rates (ASDRs) of mental disorders among people over 60 years in relation to the Socio-demographic Index (SDI) across 204 countries and territories, 2021. (A) Age-standardized incidence rate; (B) Age-standardized DALY rate.


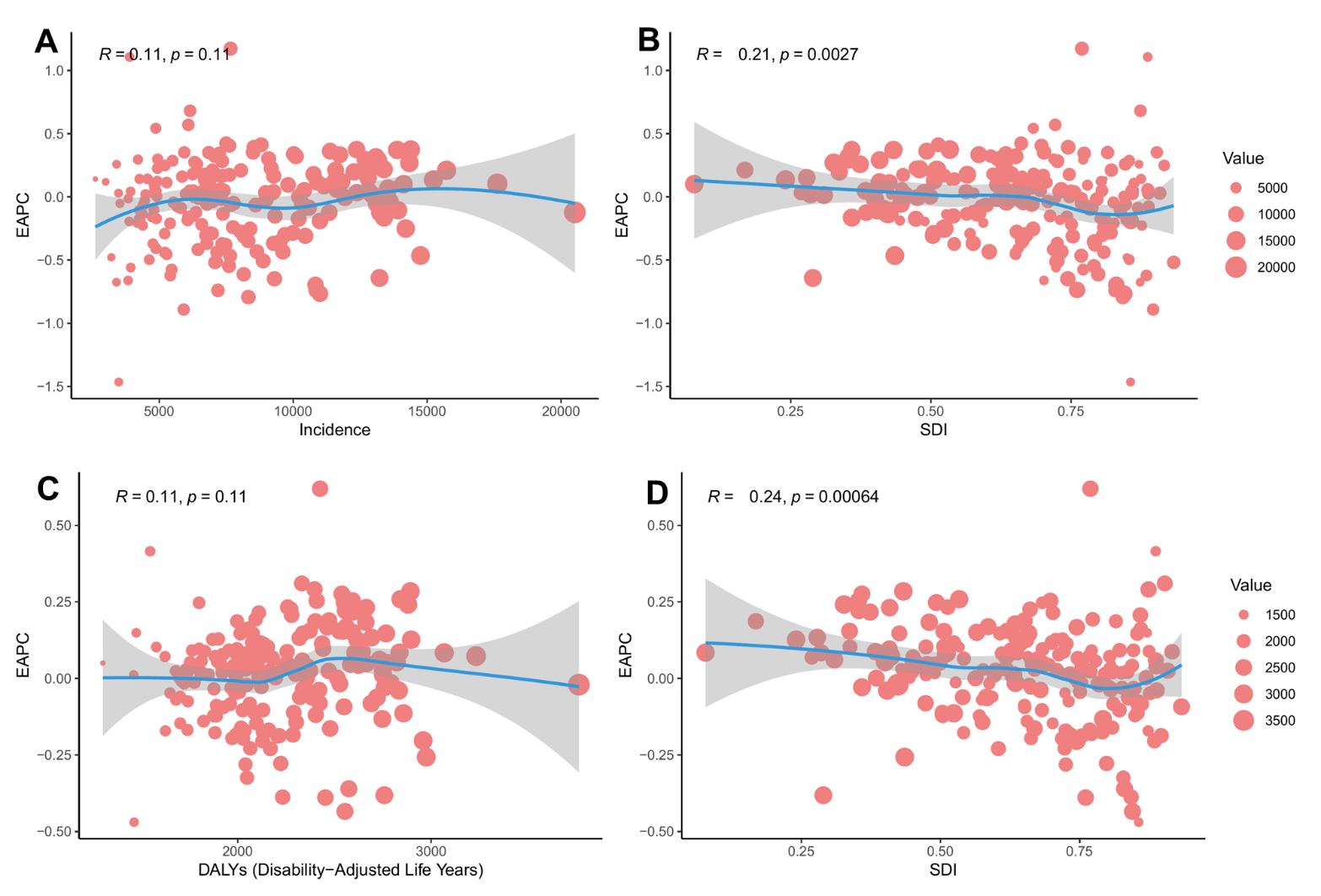


Figure S2.​​ Association between age-standardized rates of mental disorders and the Socio-demographic Index (SDI) among people over 60 years across 204 countries in 2021. (A) Incidence rates versus estimated annual percentage change (EAPC); (B) SDI versus EAPC of incidence; (C) Disability-adjusted life years (DALYs) versus EAPC; (D) SDI versus EAPC of DALYs.


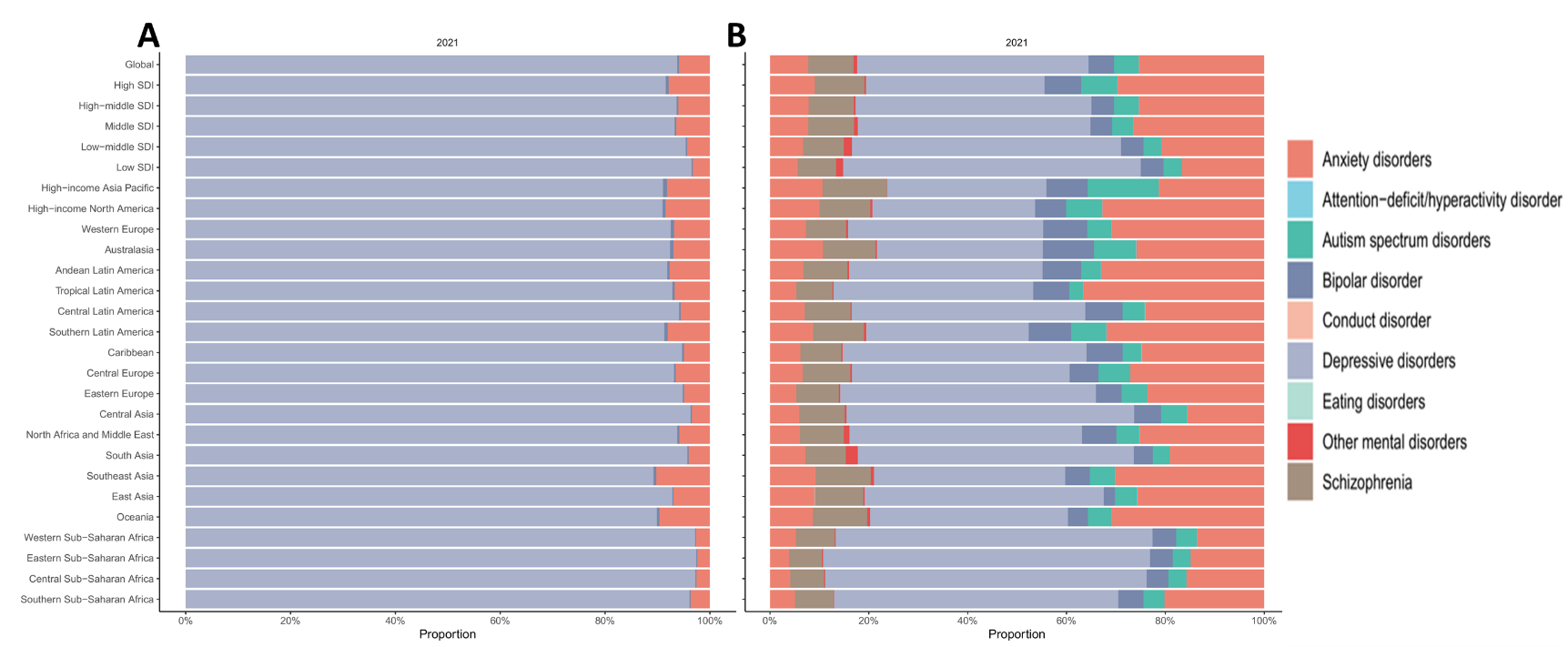


Figure S3: The proportional distribution of age-standardized incidence rates(A) and DALY rates(B) for nine mental disorders among people over 60 years.


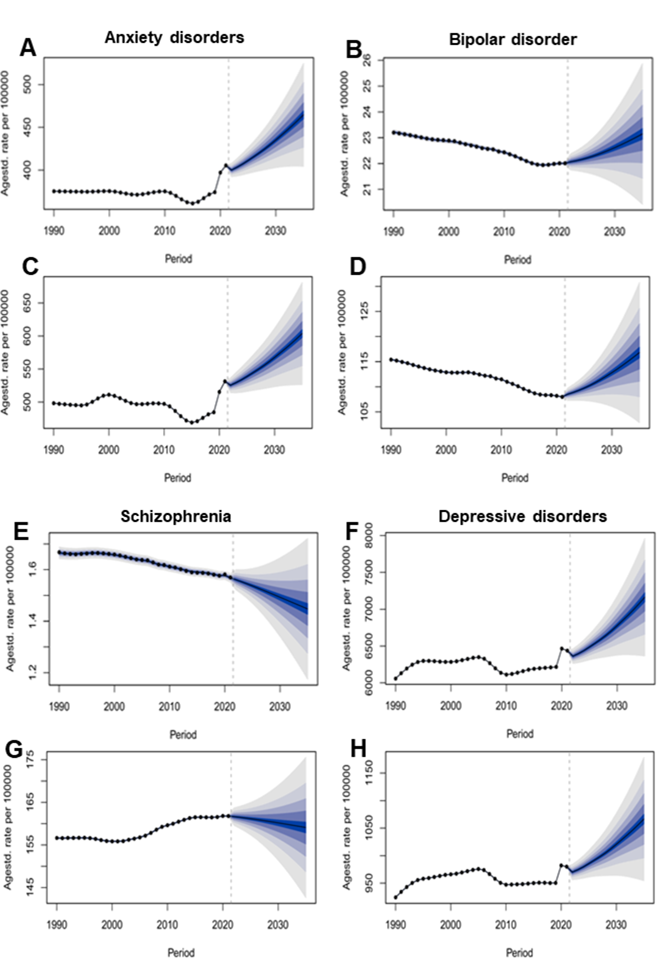


Figure S4: Global projections of age-standardized incidence rate (ASIR) (ABEF)and age-standardized disability-adjusted life year rate (ASDR)(CDGH) of Anxiety, Bipolar, Schizophrenia, Depression among people over 60 years for both sexes from 1990 to 2035.

Table S1: Results of Slope Coefficients for Health Inequality Analysis among people over 60 years.

| Year | Variable | Slope-Coefficient（95%CI） |
| --- | --- | --- |
| 1990 | Incidence | -5587.34(-6644.69, -4529.99) |
| 2021 | Incidence | -6047.42(-7152.38, -4942.45) |
| 1990 | DALYs | -408.64(-548.36, -268.91) |
| 2021 | DALYs | -440.72(-588.50, -292.94) |

DALY: disability-adjusted life year.

Table S2: Bayesian age-period-cohort (BAPC) projections of age-standardized incidence rates (ASIR) and disability-adjusted life year rates (ASDR) (per 100,000 population) for mental disorders among people over 60 years, 2025-2040.

|  | Both | | Male | | Female | |
| --- | --- | --- | --- | --- | --- | --- |
|  | ASIR | ASDR | ASIR | ASDR | ASIR | ASDR |
| 2021 | 6866.46 | 2093.83 | 5556.28 | 1848.96 | 8000.19 | 2305.82 |
| 2022 | 6780.30 | 2072.37 | 5478.12 | 1832.08 | 7897.59 | 2279.46 |
| 2023 | 6818.82 | 2080.94 | 5515.22 | 1839.46 | 7937.72 | 2288.69 |
| 2024 | 6865.84 | 2091.51 | 5559.85 | 1848.41 | 7987.72 | 2300.21 |
| 2025 | 6920.50 | 2103.97 | 5611.47 | 1858.85 | 8046.37 | 2313.89 |
| 2026 | 6977.11 | 2116.79 | 5665.72 | 1869.65 | 8106.43 | 2327.95 |
| 2027 | 7035.36 | 2129.64 | 5721.44 | 1880.50 | 8168.10 | 2342.06 |
| 2028 | 7098.86 | 2143.49 | 5781.96 | 1892.16 | 8235.66 | 2357.30 |
| 2029 | 7169.44 | 2158.83 | 5848.94 | 1905.01 | 8311.22 | 2374.24 |
| 2030 | 7246.47 | 2175.60 | 5921.99 | 1918.98 | 8393.91 | 2392.82 |
| 2031 | 7325.65 | 2192.75 | 5997.69 | 1933.30 | 8478.43 | 2411.80 |
| 2032 | 7406.65 | 2210.02 | 6075.16 | 1947.72 | 8564.76 | 2430.93 |
| 2033 | 7492.04 | 2228.04 | 6156.79 | 1962.77 | 8655.87 | 2450.92 |
| 2034 | 7583.07 | 2247.15 | 6243.74 | 1978.67 | 8753.18 | 2472.14 |
| 2035 | 7679.28 | 2267.30 | 6335.70 | 1995.38 | 8856.09 | 2494.53 |

Table S3: Age-standardized rates (per 100,000 population) and absolute case counts (in hundreds) of incident mental disorders among people over 60 years across 204 countries and territories, 1990-2021.

|  | 1990 | | 2021 | | EAPC(95%CI) |
| --- | --- | --- | --- | --- | --- |
|  | Numbers *10^2^ (95%UI) | ASPR(95%UI) | Numbers *10^2^ (95%UI) | ASPR(95%UI) |  |
| Mongolia | 112.19 [83.84-149.52] | 9311.34 [6924.55-12459.48] | 242.48 [167.13-338.26] | 9811.27 [6723.3-13769.88] | 0.1 [0.06 to 0.13] |
| Montenegro | 51.62 [38.57-68.83] | 6742.62 [5038.2-8985.14] | 97.38 [67.69-136.76] | 7322.74 [5070.39-10312.86] | -0.02 [-0.11 to 0.08] |
| Russian Federation | 17289.2 [13229.19-22157.32] | 7390.67 [5674.7-9451.21] | 23631.89 [18137.52-30238.87] | 7160.4 [5478.03-9185.9] | -0.44 [-0.57 to -0.31] |
| Taiwan (Province of China) | 781.11 [608.62-1003.58] | 3982.64 [3073.06-5149.96] | 2433.28 [1770.97-3299.79] | 4269.7 [3099.88-5797.73] | 0.14 [0.12 to 0.17] |
| Ukraine | 11003.82 [8365.04-14183.2] | 11532.4 [8786.35-14845.45] | 11539.88 [8332.35-15537.04] | 10873.5 [7845.73-14649.61] | -0.74 [-0.88 to -0.59] |
| Solomon Islands | 6.49 [5.01-8.49] | 4343.76 [3307.98-5731.78] | 16.24 [11.75-22.56] | 4492.7 [3223.42-6281.59] | 0.02 [-0.02 to 0.06] |
| Romania | 2168.2 [1656.95-2814.15] | 5999.58 [4577.37-7796.26] | 3235.65 [2292.74-4535.12] | 6470.61 [4589.78-9070.11] | -0.01 [-0.11 to 0.08] |
| Malaysia | 634.2 [479.38-837.15] | 6036.44 [4551.03-7985.74] | 2156.5 [1509.2-2979.71] | 6096.88 [4247.66-8453.15] | -0.16 [-0.34 to 0.02] |
| Region |  |  |  |  |  |
| Canada | 1497.09 [1174.89-1901.99] | 3544.23 [2779.08-4509.48] | 3252.76 [2282.85-4564.62] | 3406.91 [2391.64-4779.16] | -0.68 [-0.94 to -0.41] |
| Bosnia and Herzegovina | 358.67 [270.22-475.89] | 7542.75 [5652.64-10040.76] | 602.87 [415.79-855.66] | 7122.16 [4899.59-10131.47] | -0.51 [-0.64 to -0.39] |
| Cook Islands | 0.67 [0.48-0.9] | 4558.26 [3236.66-6211.56] | 1.64 [1.12-2.38] | 4822.59 [3275.12-6988.51] | 0.09 [0.06 to 0.12] |
| Republic of Korea | 1003.85 [776.84-1306.4] | 3096.5 [2376.45-4055.32] | 4844.42 [3512.31-6579.44] | 3896.22 [2819.89-5295.81] | 1.11 [0.69 to 1.52] |
| Democratic People's Republic of Korea | 986.68 [758.04-1298.4] | 5536.08 [4210.42-7316.68] | 2059.72 [1453.26-2899.94] | 5206.96 [3673.41-7333.89] | -0.29 [-0.32 to -0.26] |
| Dominican Republic | 343.4 [255.01-461.19] | 7951.83 [5894.23-10690.25] | 1101.18 [763.64-1550.89] | 9086.91 [6298.14-12805.17] | 0.3 [0.14 to 0.47] |
| Japan | 7072.46 [5726.55-8742.26] | 3271.7 [2648.03-4045.55] | 15491.75 [12435.01-19434.19] | 3412.32 [2758.32-4257.94] | 0.26 [0.15 to 0.36] |
| Turkmenistan | 191.36 [143.65-253.34] | 8682.74 [6495.8-11492.01] | 388.49 [275.29-542.25] | 8397.89 [5908.53-11794.41] | -0.25 [-0.34 to -0.17] |
| Democratic Republic of the Congo | 2121.93 [1576.91-2820.47] | 12462.26 [9164.25-16655.65] | 4891.43 [3420.93-6904.15] | 12807.4 [8896.99-18159.54] | 0.05 [0.01 to 0.08] |
| Grenada | 5.94 [4.44-8.06] | 6286.46 [4724.71-8500.06] | 9.25 [6.48-13.19] | 6664.84 [4658.62-9499.68] | 0.05 [-0.03 to 0.13] |
| Puerto Rico | 252.05 [191.5-329.45] | 5445.1 [4137.52-7124.5] | 515.82 [361.19-725.43] | 5537.44 [3901.9-7740.86] | -0.04 [-0.14 to 0.06] |
| Georgia | 775.58 [583.13-1027.94] | 9714.03 [7303.04-12881.16] | 843.93 [586.32-1163.8] | 10409.77 [7223.43-14366.23] | 0.05 [-0.01 to 0.12] |
| Palestine | 116.59 [85.87-156.71] | 11093.08 [8146.37-14952.1] | 334.79 [233.83-473.95] | 11631.59 [8092.89-16495.24] | 0.06 [-0.05 to 0.16] |
| Jamaica | 131.67 [97.75-177.98] | 5746.66 [4276.37-7763.77] | 245.95 [175.01-350.02] | 6262 [4467.38-8888.03] | 0.18 [0.07 to 0.29] |
| Guam | 4 [3.06-5.22] | 4601.59 [3479.93-6053.95] | 13.66 [9.64-18.98] | 4929.97 [3478.99-6851.71] | 0.12 [0.06 to 0.18] |
| Central African Republic | 157.06 [116.84-210.24] | 13137.61 [9667.77-17693.58] | 292.22 [200.44-404.22] | 13455.19 [9164.16-18756.55] | 0.01 [-0.02 to 0.05] |
| United States Virgin Islands | 6.22 [4.68-8.35] | 6380.12 [4780.31-8583.69] | 17.53 [12.04-24.73] | 6989.63 [4812.59-9843.73] | 0.21 [0.14 to 0.28] |
| Brunei Darussalam | 2.59 [1.99-3.4] | 2453.36 [1877.04-3238.62] | 10.24 [7.35-14.27] | 2620.67 [1867.94-3678.43] | 0.14 [0.06 to 0.22] |
| Lao People's Democratic Republic | 94.32 [73.22-124.44] | 3921.75 [3014.96-5202.82] | 205.09 [150.11-282.08] | 3885.08 [2816.9-5364.45] | -0.19 [-0.26 to -0.13] |
| Tuvalu | 0.39 [0.28-0.53] | 4691.28 [3339.09-6436.37] | 0.64 [0.43-0.93] | 4849.53 [3259.4-7077.35] | 0.03 [0 to 0.05] |
| Antigua and Barbuda | 4 [2.94-5.5] | 5822.27 [4294.37-7986] | 8.25 [5.83-11.48] | 6145.05 [4324.74-8569.21] | 0.05 [-0.09 to 0.18] |
| United States of America | 17223.34 [13950.65-21447.94] | 4119.43 [3341.03-5128.93] | 37884.71 [30627.39-47361.57] | 4843.16 [3916.3-6053.72] | 0.11 [-0.02 to 0.24] |
| Hungary | 1625.52 [1222.03-2141.26] | 8297.2 [6242.98-10933.28] | 1988.9 [1374.12-2765.29] | 7606.18 [5255.29-10563.94] | -0.55 [-0.76 to -0.33] |
| Saint Lucia | 7.11 [5.35-9.46] | 6317.45 [4742.09-8417.92] | 20.88 [14.61-29.39] | 6902.48 [4824.37-9716.79] | 0.09 [-0.01 to 0.19] |
| North Macedonia | 145.67 [109.99-191.36] | 6418.75 [4828.77-8450.45] | 291.7 [208.55-403.89] | 6590.23 [4687.22-9166.28] | -0.22 [-0.32 to -0.12] |
| American Samoa | 0.92 [0.71-1.2] | 3756.22 [2876.2-4925.92] | 2.18 [1.59-3] | 3846.57 [2780.45-5309.27] | -0.01 [-0.06 to 0.03] |
| Northern Mariana Islands | 0.53 [0.42-0.69] | 3696.64 [2840.04-4822.96] | 2.59 [1.88-3.62] | 4209.81 [3017.1-5900.53] | 0.3 [0.25 to 0.34] |
| Saint Vincent and the Grenadines | 5.8 [4.38-7.73] | 6297.68 [4742.92-8404.51] | 13.01 [9.1-18.05] | 6978.06 [4872.41-9695.84] | 0.2 [0.12 to 0.27] |
| Australia | 1304.75 [1017.01-1679.48] | 5088.58 [3960.95-6558.16] | 2863.66 [1982.76-4044.24] | 4914.14 [3403.91-6937.69] | 0.01 [-0.18 to 0.2] |
| United Republic of Tanzania | 1606.25 [1206.52-2115.97] | 12901.84 [9622.69-17101.58] | 3661.29 [2564.94-5158.89] | 13119.37 [9149.85-18516.28] | -0.05 [-0.09 to -0.02] |
| New Zealand | 188.73 [141.99-247.05] | 3671.52 [2761.13-4811.06] | 352.11 [262.79-474.43] | 3213.75 [2400.79-4331.02] | -0.48 [-0.64 to -0.31] |
| Syrian Arab Republic | 382.17 [284.54-515.71] | 6279.92 [4661.65-8486.9] | 1095.27 [764.32-1590.45] | 6688.74 [4641.98-9717.37] | 0.13 [0.06 to 0.21] |
| Barbados | 23.19 [17.33-30.95] | 6104.09 [4582.71-8104.92] | 46.51 [32.44-63.6] | 6675.85 [4650.47-9137.96] | 0.18 [0.1 to 0.26] |
| Republic of Moldova | 518.05 [383.63-678.48] | 9218.93 [6827.11-12084.57] | 700.39 [487.1-992.18] | 8648.15 [6002.36-12274.05] | -0.43 [-0.54 to -0.33] |
| Burkina Faso | 627.84 [470.54-827.87] | 12634.24 [9409.88-16710.99] | 1321.65 [932.09-1853.95] | 12938.16 [9083.29-18208.67] | 0.02 [-0.05 to 0.09] |
| Belize | 6.91 [5.22-9.31] | 6067.13 [4587.6-8160.28] | 23.3 [16.38-32.37] | 6711.01 [4705.36-9329.18] | 0.18 [0.1 to 0.26] |
| Ireland | 297.33 [227.4-392.4] | 5586.54 [4272.36-7388.74] | 618.94 [434.93-871.76] | 6153.57 [4328.92-8674.29] | 0.68 [0.54 to 0.83] |
| United Arab Emirates | 21.19 [15.84-28.7] | 6031.21 [4498.3-8165.38] | 221.38 [155.54-317.32] | 6067.11 [4207.43-8724.2] | -0.18 [-0.25 to -0.11] |
| Tokelau | 0.09 [0.06-0.12] | 4605.06 [3270.92-6312.57] | 0.09 [0.06-0.13] | 4831.23 [3249.68-6927.23] | 0.08 [0.05 to 0.1] |
| Bermuda | 5.52 [4.16-7.36] | 7085.59 [5338.11-9460.93] | 12.51 [8.63-17.85] | 6915.43 [4781.39-9860.17] | -0.28 [-0.36 to -0.21] |
| Saint Kitts and Nevis | 4.45 [3.09-6.11] | 8496.94 [5887.58-11700.05] | 8.13 [5.4-11.7] | 8971.26 [5915.34-12978.32] | 0.03 [-0.02 to 0.09] |
| Greenland | 2 [1.48-2.71] | 5343.26 [3950.39-7248.46] | 5.07 [3.53-7.08] | 5409.01 [3753.92-7582.9] | -0.21 [-0.28 to -0.14] |
| China | 47716.45 [38680.1-59324.69] | 4920.38 [3967.63-6144.85] | 163140.01 [131698.77-203098.2] | 6090.63 [4894.29-7599.61] | 0.57 [0.45 to 0.69] |
| Cambodia | 237.71 [183.39-313.72] | 4675.26 [3577.92-6203.22] | 702.81 [504.26-965.18] | 4740.15 [3375.44-6546.72] | -0.16 [-0.21 to -0.1] |
| Indonesia | 3574.55 [2840.03-4470.94] | 3267.78 [2583.57-4100.87] | 9972.31 [7824.08-12511.8] | 3485.04 [2723.15-4391.16] | 0.03 [-0.07 to 0.13] |
| Maldives | 5.55 [4.27-7.3] | 5644.86 [4291.24-7480.97] | 18.92 [13.67-26.11] | 5282.89 [3799.39-7311.71] | -0.45 [-0.53 to -0.38] |
| Myanmar | 755.43 [586.76-987.18] | 2761.24 [2124.79-3632.42] | 1793.39 [1311.71-2465.26] | 3004.36 [2181.02-4151.52] | 0.12 [-0.01 to 0.24] |
| Philippines | 1844.79 [1433.66-2350.29] | 5649.79 [4380.34-7208.44] | 5065.49 [3940.07-6391.45] | 5212.45 [4040.98-6589.41] | -0.44 [-0.53 to -0.35] |
| Sri Lanka | 536.13 [413.66-698.7] | 4308.54 [3312.08-5635.89] | 1374.29 [1001.44-1902.43] | 3841.66 [2788.71-5336.88] | -0.66 [-0.78 to -0.54] |
| Thailand | 1641.01 [1262.96-2146.59] | 4112.44 [3150.62-5395.46] | 6962.75 [4861.49-9558.04] | 4874.11 [3400.89-6694.4] | 0.54 [0.49 to 0.59] |
| Timor-Leste | 11.92 [9.15-15.74] | 4352.79 [3305.98-5785.38] | 46.25 [33.15-63.52] | 4300.11 [3069.52-5937.39] | -0.19 [-0.25 to -0.12] |
| Viet Nam | 1710.51 [1314.28-2279.32] | 3427.21 [2619.74-4579.21] | 4331.2 [3082.42-6062.62] | 3535.38 [2499.71-4972.81] | -0.05 [-0.19 to 0.08] |
| Fiji | 15.33 [11.8-20.1] | 4179.45 [3186.63-5508.8] | 42.04 [30.65-57.65] | 4567.61 [3295.05-6287.64] | 0.14 [0.1 to 0.19] |
| Kiribati | 1.83 [1.43-2.37] | 4460.06 [3434.01-5831.08] | 3.59 [2.6-4.95] | 4387.99 [3145.3-6103.45] | -0.14 [-0.19 to -0.08] |
| Marshall Islands | 0.77 [0.6-1.02] | 4250.48 [3239.39-5623.9] | 1.65 [1.19-2.27] | 4250.64 [3022.41-5936.71] | -0.11 [-0.14 to -0.07] |
| Micronesia (Federated States of) | 2.55 [1.95-3.34] | 4357.53 [3295.01-5735.61] | 3.86 [2.79-5.3] | 4390.97 [3145.95-6065.92] | -0.06 [-0.1 to -0.03] |
| Papua New Guinea | 88.18 [68.14-115.89] | 4438.69 [3377.16-5886.26] | 230.76 [164.33-321.45] | 4414.14 [3118.69-6186.44] | -0.07 [-0.09 to -0.04] |
| Samoa | 4.32 [3.3-5.63] | 4287.03 [3242.77-5627.02] | 7.11 [5.06-9.83] | 4187.03 [2964.74-5812.18] | -0.21 [-0.26 to -0.15] |
| Tonga | 2.48 [1.92-3.23] | 3783.67 [2896.1-4950.07] | 3.79 [2.7-5.29] | 3933.15 [2798.28-5494.75] | 0.04 [0 to 0.09] |
| Vanuatu | 3 [2.34-3.93] | 4350.15 [3344.42-5751.27] | 8.62 [6.29-11.74] | 4462.55 [3221.41-6118.76] | -0.03 [-0.07 to 0.01] |
| Armenia | 239.58 [178.33-319.65] | 7361.61 [5470.61-9833.52] | 508.91 [360.6-703.98] | 8506.93 [5993.43-11810.25] | 0.35 [0.28 to 0.43] |
| Azerbaijan | 396.52 [295.12-528.86] | 7061.05 [5252.19-9408.19] | 912.22 [645.32-1274.37] | 7415.19 [5195.86-10371.49] | 0.05 [-0.04 to 0.15] |
| Kazakhstan | 1427.88 [1074.78-1865.55] | 9416.98 [7069.96-12333.13] | 1933.76 [1373.05-2692.07] | 8881.82 [6257.86-12412.58] | -0.51 [-0.59 to -0.42] |
| Kyrgyzstan | 377.73 [279.81-496.8] | 10359.35 [7669.31-13647.25] | 535.47 [374.39-739.97] | 9617.38 [6691.31-13342.31] | -0.43 [-0.54 to -0.33] |
| Tajikistan | 246.52 [185.32-325.78] | 7725.61 [5790.79-10223.32] | 479.43 [338.41-665.14] | 7352.34 [5145.61-10261.63] | -0.37 [-0.48 to -0.26] |
| Uzbekistan | 1204.13 [904.33-1582.25] | 8841.97 [6631.75-11607.74] | 2507.63 [1776.8-3458.92] | 8320.23 [5845.42-11524.42] | -0.35 [-0.44 to -0.25] |
| Albania | 114.57 [87.37-152.37] | 4813.87 [3653.8-6417.61] | 315.96 [222.06-437.9] | 5282.63 [3707.55-7332.21] | 0.11 [0.03 to 0.2] |
| Bulgaria | 1290.85 [992.03-1685.72] | 7787.95 [5968.38-10182.75] | 1485.53 [1038.5-2064.56] | 7650.38 [5370.82-10619.32] | -0.47 [-0.61 to -0.32] |
| Croatia | 704.15 [524.52-920.26] | 9060.63 [6690.75-11858.28] | 979.09 [672.36-1370.39] | 8155.9 [5602.16-11405.7] | -0.61 [-0.72 to -0.5] |
| Czechia | 1522.83 [1133.94-2005.49] | 8307.48 [6190.33-10935.39] | 2028.82 [1435.04-2794.16] | 7194.94 [5095.89-9894.59] | -0.74 [-0.89 to -0.59] |
| Poland | 2178.37 [1696.55-2759.29] | 3804.97 [2964.41-4818.71] | 4159.84 [3257.62-5263.69] | 4247.21 [3317.57-5386.48] | -0.17 [-0.43 to 0.09] |
| Serbia | 1108.68 [840.85-1462.74] | 7697.97 [5773.38-10215.57] | 1682.73 [1163.68-2342.65] | 7476.89 [5176.08-10399.53] | -0.38 [-0.46 to -0.3] |
| Slovakia | 545.98 [415.5-720.42] | 6948.67 [5284.73-9170.56] | 898.1 [625.3-1250.99] | 6982.57 [4850.08-9746.68] | -0.29 [-0.49 to -0.09] |
| Slovenia | 305.81 [230.49-402.08] | 9697.19 [7322.35-12727.5] | 486.61 [336.14-682.61] | 8329.65 [5767.76-11660.36] | -0.79 [-0.89 to -0.7] |
| Belarus | 1668.88 [1233.58-2203.79] | 9824.92 [7286.71-12943.31] | 2331.17 [1639.66-3328.13] | 10585.68 [7428.04-15124.52] | -0.09 [-0.17 to 0] |
| Estonia | 327.95 [245.56-433.26] | 12290.87 [9219-16217.47] | 391.64 [270.38-548.73] | 11001.72 [7618.49-15390.39] | -0.77 [-0.91 to -0.62] |
| Latvia | 542.78 [403.72-720.08] | 11612.19 [8651.62-15378.55] | 568.59 [389.38-792.67] | 10834.1 [7444.16-15095.9] | -0.7 [-0.88 to -0.51] |
| Lithuania | 605.03 [445.26-802.9] | 10371.04 [7646.03-13740.66] | 830.48 [566.58-1168.6] | 11000.11 [7523.17-15452.3] | -0.19 [-0.29 to -0.09] |
| Singapore | 121.46 [93.47-156.56] | 4881.15 [3736.32-6316.15] | 385.09 [275.23-536.95] | 3492.62 [2485.34-4883.32] | -1.47 [-1.66 to -1.27] |
| Andorra | 4.23 [3.2-5.65] | 5748.87 [4332.67-7715.01] | 11.25 [7.75-16.02] | 5809.64 [4008.17-8249.74] | -0.09 [-0.18 to 0] |
| Austria | 787.91 [595.82-1030.21] | 5059.66 [3835.05-6603.6] | 1055.06 [744.23-1504.43] | 4631.67 [3286.28-6578.96] | -0.5 [-0.6 to -0.4] |
| Belgium | 1036.58 [861.64-1243.62] | 5097.46 [4238.49-6114.3] | 1629.65 [1136.3-2298.9] | 5500.35 [3848.68-7748.27] | 0.29 [0.15 to 0.42] |
| Cyprus | 50.25 [38.04-67.67] | 4754.75 [3593.06-6423.23] | 130.06 [90.9-182.47] | 4803.87 [3356.61-6747.24] | -0.1 [-0.19 to -0.02] |
| Denmark | 761.46 [566.94-1024.82] | 7268.04 [5431.04-9793.45] | 897.05 [620.74-1283.27] | 5915.86 [4107.38-8449.33] | -0.89 [-1.08 to -0.7] |
| Finland | 552.51 [424.56-726.45] | 5940.39 [4568.84-7806.77] | 972.05 [671.64-1368.99] | 6002.11 [4154.91-8427.49] | 0.02 [-0.14 to 0.19] |
| France | 8058.15 [6647.63-9637.11] | 7411.98 [6131.77-8849.69] | 11347.7 [7844.52-15757.37] | 6366.42 [4407.96-8820.7] | -0.12 [-0.42 to 0.18] |
| Germany | 8941.77 [7268.84-11044.27] | 5469.49 [4453-6743.15] | 14581.77 [10354.92-20321.45] | 5939.03 [4247.67-8251.36] | 0.35 [0.19 to 0.51] |
| Greece | 1904.77 [1430.44-2554.71] | 9543.72 [7169.99-12798.07] | 2815.93 [1929.91-3964.5] | 9510.6 [6565.75-13332.37] | -0.36 [-0.64 to -0.07] |
| Iceland | 17.1 [13.11-22.16] | 4617.64 [3546.97-5978.08] | 33.43 [23.72-46.57] | 4390.3 [3123.66-6103.04] | -0.23 [-0.34 to -0.12] |
| Israel | 517.76 [385.25-687.4] | 8157.9 [6080.23-10833.98] | 1233.54 [867.24-1730.04] | 7756.58 [5455.31-10891.98] | -0.24 [-0.32 to -0.16] |
| Italy | 8114.64 [6227.24-10403.33] | 6880.72 [5288.32-8814.75] | 12187.56 [9225.61-15701.19] | 6737.7 [5117.3-8659.38] | -0.3 [-0.4 to -0.21] |
| Luxembourg | 43.71 [32.5-57.88] | 6145.52 [4581.97-8120.92] | 72.33 [51.52-101.44] | 5413.84 [3867.65-7585.62] | -0.62 [-0.74 to -0.51] |
| Malta | 26.94 [20.44-36.07] | 4942.59 [3742.74-6625.17] | 63.85 [45.04-91.01] | 4896.46 [3466.87-6972.04] | -0.17 [-0.27 to -0.08] |
| Netherlands | 1477 [1214.97-1786.19] | 5716.22 [4705.13-6907.14] | 2595.54 [1812.38-3743.35] | 5683.84 [3971.91-8197.87] | -0.08 [-0.21 to 0.06] |
| Norway | 434.46 [336.18-554.77] | 4905.8 [3797.93-6269.23] | 661.95 [500.43-859.39] | 5216.32 [3950.19-6764.25] | 0.25 [0.14 to 0.35] |
| Portugal | 1787.73 [1330.25-2376.65] | 9627.96 [7163.97-12814.56] | 2928.13 [2046.94-4118.62] | 9303.21 [6513.47-13098.32] | -0.65 [-0.94 to -0.36] |
| Spain | 4870.33 [4081.93-5760.93] | 6692.17 [5601.56-7930.9] | 9283.61 [6605.2-12980.93] | 7665.88 [5487.7-10661.5] | 1.17 [0.89 to 1.45] |
| Sweden | 1319.19 [1029.99-1686.44] | 6710.65 [5241.72-8592.61] | 1786.41 [1340.74-2420.35] | 6684.29 [5043.49-9021.55] | 0.06 [-0.02 to 0.13] |
| Switzerland | 912 [682.63-1208.87] | 6878.9 [5167.79-9111.18] | 1594.16 [1099.87-2279.21] | 7083.03 [4908.79-10101.12] | -0.52 [-0.94 to -0.09] |
| United Kingdom | 8080.53 [6234.44-10387.97] | 6822.6 [5269.39-8780.17] | 11586.26 [8899.49-14909.35] | 7123.67 [5484.59-9165.2] | 0.05 [-0.03 to 0.13] |
| Argentina | 1791.78 [1355.79-2357.95] | 4316.11 [3259.31-5689.62] | 2825.46 [2083.79-3797.89] | 3940.38 [2910.27-5289.19] | -0.56 [-0.67 to -0.45] |
| Chile | 753.14 [594.44-949.52] | 6148.13 [4835.7-7771.36] | 1815.96 [1264.65-2587.71] | 5464.8 [3806.76-7783.85] | -0.58 [-0.7 to -0.45] |
| Uruguay | 218.15 [163.17-291.09] | 4222.88 [3159.8-5635.29] | 350.38 [249.3-495.26] | 4947.1 [3535.93-6985.01] | 0.3 [0.2 to 0.4] |
| Bahamas | 10.34 [7.76-13.93] | 5907.68 [4427.44-7963.77] | 31.22 [21.92-44.41] | 6272.92 [4391.76-8960.96] | 0.05 [-0.07 to 0.17] |
| Cuba | 1279.63 [959.47-1709.11] | 9991.05 [7489.08-13342.81] | 2352.02 [1647.66-3255.2] | 9692.38 [6815.71-13394.06] | -0.3 [-0.41 to -0.19] |
| Dominica | 4.6 [3.43-6.17] | 5992.24 [4476.05-8048.14] | 6.84 [4.83-9.75] | 6330.09 [4452.77-9036.48] | 0 [-0.1 to 0.1] |
| Guyana | 38.47 [29.07-51.4] | 8726.93 [6567.7-11689.03] | 80.04 [56.49-113.06] | 10104.68 [7094.17-14315.01] | 0.32 [0.26 to 0.38] |
| Haiti | 255.97 [192.23-343.89] | 6870.93 [5105.89-9273.28] | 593.13 [424.91-835.14] | 7303.81 [5202.89-10355.55] | 0.06 [-0.02 to 0.14] |
| Suriname | 25.63 [19.05-34.3] | 8500.68 [6293.98-11382.76] | 78.79 [54.82-110.71] | 10024.62 [6977.56-14088.51] | 0.34 [0.26 to 0.41] |
| Trinidad and Tobago | 75.11 [56.63-99.21] | 7255.96 [5458.33-9606.82] | 201.88 [141.66-284.5] | 7773.28 [5444.71-10972.9] | -0.05 [-0.15 to 0.05] |
| Bolivia (Plurinational State of) | 302.19 [224.91-399.18] | 8107.2 [6003.94-10756.96] | 968.69 [672.8-1365.33] | 8597.58 [5943.93-12154.03] | -0.06 [-0.17 to 0.04] |
| Ecuador | 390.24 [299.45-516.4] | 6336 [4850.78-8395.83] | 1518.83 [1049.74-2073.57] | 7500.69 [5182.01-10245.78] | 0.42 [0.28 to 0.57] |
| Peru | 660.33 [506.96-870.55] | 4761.18 [3646.17-6283.71] | 1943.83 [1406.18-2701.86] | 4769.31 [3451.02-6628.68] | -0.37 [-0.53 to -0.21] |
| Colombia | 1018.04 [777.5-1336.15] | 5036.04 [3829.28-6625.43] | 3410.8 [2417.84-4694.19] | 4878.24 [3461.11-6708.31] | -0.41 [-0.57 to -0.24] |
| Costa Rica | 140.37 [105.83-187.16] | 6736.41 [5073.85-8983.53] | 518.61 [358.09-727.85] | 7377.11 [5094.19-10347.29] | 0.17 [0.11 to 0.23] |
| El Salvador | 257.92 [191.89-345.21] | 7283.69 [5414.94-9751.54] | 567.96 [393.06-815.29] | 7380.16 [5128.22-10562.27] | -0.13 [-0.22 to -0.04] |
| Guatemala | 348.81 [266.54-459.02] | 8834.45 [6691.05-11706.35] | 1243.93 [882.4-1733.11] | 9323.64 [6594.53-13013.28] | -0.15 [-0.47 to 0.17] |
| Honduras | 171.72 [128.47-229.43] | 7387.9 [5500.97-9899.26] | 661.15 [460.18-931.59] | 8807.84 [6094.58-12450.48] | 0.41 [0.34 to 0.48] |
| Mexico | 3416.33 [2721.77-4294.25] | 6990.96 [5558.63-8795.76] | 12083.65 [9528.19-15236.45] | 7788.81 [6133.66-9827.31] | -0.05 [-0.21 to 0.11] |
| Nicaragua | 113.4 [84.45-152.57] | 6531.71 [4849.18-8794.6] | 417.28 [289.4-590.85] | 7202.99 [4983.71-10215.67] | 0.17 [0.1 to 0.23] |
| Panama | 118.68 [88.34-158.61] | 6724.3 [5002.57-8991.56] | 384.03 [268.78-545.41] | 7002.06 [4908.91-9933.81] | -0.04 [-0.11 to 0.04] |
| Venezuela (Bolivarian Republic of) | 835.82 [637.44-1103.71] | 7489.32 [5693.89-9914.23] | 2924.98 [2039.36-4127.24] | 7684.43 [5353.66-10871.35] | -0.06 [-0.11 to -0.01] |
| Brazil | 8527.18 [6867.49-10644.5] | 8042.34 [6453.33-10066.6] | 25547.64 [20325.92-32082.87] | 8037.35 [6384.35-10101.8] | -0.28 [-0.37 to -0.18] |
| Paraguay | 146.24 [111.1-196.5] | 5521.67 [4189.5-7425.21] | 454.29 [318.73-638.11] | 6324.21 [4429.29-8888.89] | 0.27 [0.13 to 0.42] |
| Algeria | 1023.31 [766.42-1370.22] | 6808.31 [5080.4-9140.5] | 2997.21 [2096.32-4253.16] | 6953.94 [4842.59-9881.32] | -0.04 [-0.09 to 0.02] |
| Bahrain | 15.11 [11.42-19.95] | 8294.7 [6223.42-11014.55] | 83.53 [59.4-118.17] | 8161.65 [5719.61-11592.67] | -0.31 [-0.4 to -0.23] |
| Egypt | 1832.53 [1376.99-2467.75] | 5900.89 [4403.16-7978.95] | 5436.92 [3848.53-7436.81] | 7075.99 [4956.48-9772.05] | 0.32 [0.21 to 0.43] |
| Iran (Islamic Republic of) | 2232.2 [1693.08-2951.81] | 6998.54 [5260.62-9272.42] | 7142.35 [5392.6-9456.14] | 7620.58 [5738.49-10102.89] | 0.41 [0.34 to 0.48] |
| Iraq | 502.36 [374.96-679.33] | 5514.87 [4110.84-7458.37] | 1586.27 [1116.62-2279.09] | 5859.12 [4105.68-8419.54] | 0.16 [0.08 to 0.24] |
| Jordan | 101.72 [76.61-134.24] | 7159.55 [5347.31-9499.54] | 560.57 [385.88-788.87] | 6777.35 [4656.83-9577.92] | -0.37 [-0.43 to -0.3] |
| Kuwait | 35.51 [26.53-47.94] | 5992.65 [4462.78-8100.48] | 185.87 [129.42-267.04] | 6402.94 [4439.81-9211.71] | 0.26 [0.22 to 0.3] |
| Lebanon | 197.11 [150.47-262.25] | 7380.08 [5586.98-9857] | 616.82 [422-881.74] | 8536.54 [5854.61-12185.21] | 0.39 [0.23 to 0.56] |
| Libya | 145.64 [108.98-198.95] | 6651.51 [4969.48-9082.81] | 412.2 [284.93-580.77] | 7357.58 [5084.6-10360.98] | 0.28 [0.24 to 0.32] |
| Morocco | 1511.92 [1132.51-2035.23] | 8786.12 [6561.38-11849.86] | 4013.61 [2799.07-5635.63] | 9269.27 [6448.81-13043.17] | 0.04 [-0.01 to 0.09] |
| Oman | 45.91 [34.21-62.58] | 6446.07 [4788.81-8790.95] | 145.28 [100.36-203.9] | 7242.89 [4987.25-10206.13] | 0.34 [0.31 to 0.38] |
| Qatar | 5.77 [4.33-7.83] | 6613.74 [4930.36-8982.53] | 54.85 [38.24-76.5] | 6783.43 [4711.32-9479.1] | -0.06 [-0.11 to 0] |
| Saudi Arabia | 385.96 [287.68-521.45] | 6165.62 [4582.24-8335.29] | 1272.84 [890.37-1773.86] | 6851.54 [4763.96-9585.37] | 0.35 [0.31 to 0.4] |
| Tunisia | 614.32 [460.6-816.43] | 10072.49 [7515.17-13449] | 1909.82 [1339.66-2660.97] | 11153.25 [7782.95-15596.3] | 0.09 [-0.01 to 0.18] |
| Turkey | 2553.65 [1992.15-3253.88] | 6334.88 [4909.48-8103.64] | 7434.84 [5125.94-10456.09] | 6246.78 [4295.06-8798.44] | -0.15 [-0.23 to -0.07] |
| Yemen | 406.74 [304.54-550] | 7204.7 [5345.93-9789.84] | 1215.19 [840.47-1750.72] | 7623.89 [5258.21-10990.74] | 0.17 [0.15 to 0.2] |
| Afghanistan | 623.08 [465.59-836.47] | 7304.6 [5418.95-9838.69] | 642.04 [449.6-919.38] | 8052.56 [5615.17-11543.96] | 0.21 [0.17 to 0.25] |
| Bangladesh | 5450.71 [4111.13-7256.63] | 10157.57 [7647.37-13549.5] | 19353.26 [13712.37-27290.9] | 11383.94 [8049.1-16078.48] | 0.36 [0.28 to 0.44] |
| Bhutan | 28.23 [21-37.53] | 10609.95 [7812.08-14172.12] | 74.16 [51.75-104.85] | 10203.93 [7110.87-14435.52] | -0.17 [-0.24 to -0.11] |
| India | 46646.26 [37100.21-58817.69] | 8905.25 [7037.15-11257.23] | 135544.68 [108638.26-170915.87] | 9269.06 [7395.44-11709.74] | -0.37 [-0.57 to -0.16] |
| Nepal | 1265.39 [950.82-1675.2] | 11982.16 [8929.25-15937.39] | 4000.82 [2710.74-5601.83] | 13879.64 [9374.44-19485.22] | 0.37 [0.13 to 0.61] |
| Pakistan | 6823.56 [5219.11-8799.77] | 10343.67 [7887.57-13371.84] | 13526.4 [9982.87-17817.86] | 9990.04 [7357.84-13191.77] | -0.29 [-0.39 to -0.18] |
| Angola | 518.05 [384.83-693.29] | 13078.5 [9636.5-17609.18] | 1641.89 [1151.48-2308.38] | 13500.63 [9362.74-19015.8] | 0.01 [-0.03 to 0.05] |
| Congo | 160.45 [118.49-213.43] | 13334.01 [9750.99-17815.49] | 365.39 [248.27-509.9] | 13242.64 [8951.04-18599.11] | -0.13 [-0.17 to -0.08] |
| Equatorial Guinea | 27.49 [20.52-37.09] | 13101.54 [9678.35-17733.94] | 70.15 [47.97-100.21] | 13544.73 [9229.6-19404.04] | 0.07 [0.01 to 0.12] |
| Gabon | 91 [67.57-121.77] | 13224.34 [9759.03-17745.75] | 160.17 [110.36-227.04] | 13600.76 [9315.67-19338.25] | -0.04 [-0.08 to 0] |
| Burundi | 394.7 [297.26-518.85] | 14838.41 [11129.47-19573.01] | 700.77 [495.42-966.79] | 13223.53 [9288.22-18315.28] | -0.64 [-0.73 to -0.55] |
| Comoros | 25.58 [19.4-33.99] | 11554.28 [8664.21-15454.69] | 66.18 [45.88-92.87] | 11934.09 [8241.73-16803.18] | 0 [-0.11 to 0.1] |
| Djibouti | 15.66 [11.87-20.95] | 11930.22 [8920.04-16057.55] | 80.69 [56.74-112.18] | 12379.98 [8608.47-17284.22] | 0.03 [-0.01 to 0.08] |
| Eritrea | 142.3 [106.79-189.08] | 13933.12 [10293.76-18626.3] | 387.18 [266.45-543.5] | 13916.49 [9495.44-19616.33] | -0.11 [-0.14 to -0.07] |
| Ethiopia | 2926.55 [2263.97-3745] | 13556.88 [10424.52-17409.58] | 6382.62 [4789.04-8260.31] | 13390.43 [10035.79-17369.12] | -0.16 [-0.3 to -0.03] |
| Kenya | 1326.86 [1059.03-1660.66] | 14371.91 [11420.8-18035.76] | 3609.46 [2875.26-4508.47] | 14201.36 [11236.35-17806.41] | -0.25 [-0.35 to -0.15] |
| Madagascar | 751.02 [556.68-995.51] | 13048.64 [9602.56-17394.22] | 1567.9 [1106.43-2174.54] | 13537.83 [9449.31-18875.32] | -0.02 [-0.07 to 0.03] |
| Malawi | 484.87 [367.52-635.69] | 11122.04 [8336.5-14685.78] | 923.44 [651.89-1262.79] | 11347.85 [7974.68-15648.72] | -0.12 [-0.2 to -0.05] |
| Mauritius | 59.83 [45.49-78.71] | 6738.74 [5078.6-8926.84] | 159.06 [112.61-222.55] | 6448.24 [4543.97-9047.16] | -0.42 [-0.62 to -0.21] |
| Mozambique | 837.38 [628.78-1110.85] | 12743.85 [9463-16986.69] | 1715.77 [1196.17-2386.89] | 14337.14 [9947.75-20055.08] | 0.27 [0.24 to 0.3] |
| Rwanda | 501.41 [378.6-660.77] | 15713.8 [11710.82-20873.12] | 1056.01 [744.56-1480.15] | 14752.38 [10284.28-20861.96] | -0.46 [-0.55 to -0.38] |
| Seychelles | 3.01 [2.3-4.02] | 4168.21 [3173.5-5554.24] | 6.1 [4.39-8.43] | 4234.52 [3030.17-5863.26] | -0.18 [-0.27 to -0.1] |
| Somalia | 283.68 [211.92-380.01] | 12942.2 [9585-17410.58] | 901.57 [637.07-1265.94] | 14119.4 [9858.46-19984.61] | 0.1 [0.05 to 0.15] |
| Uganda | 1429.83 [1080.04-1865.48] | 19655.15 [14752.06-25753.05] | 3238.95 [2299.78-4489.57] | 20513.24 [14496-28497.12] | -0.12 [-0.23 to -0.02] |
| Zambia | 324.32 [244.56-429.07] | 10639.92 [7965.07-14133.86] | 825.69 [575.52-1141.29] | 11554.83 [7997.26-16039.24] | 0.13 [0.04 to 0.21] |
| Botswana | 60.52 [45.11-81.95] | 9563.99 [7062.44-13039.53] | 175.64 [120.26-250.21] | 10761.66 [7314.12-15393.05] | 0.18 [0.11 to 0.24] |
| Lesotho | 142.1 [105.33-189.82] | 14426.55 [10643.69-19340.61] | 197.83 [135.85-280.33] | 15724.19 [10735.01-22345.61] | 0.21 [0.13 to 0.29] |
| Namibia | 61.84 [46.42-82.39] | 8210.77 [6095.38-10992.26] | 138.68 [96.15-199.5] | 8989.28 [6189.19-12989.57] | -0.02 [-0.14 to 0.1] |
| South Africa | 2341.98 [1872.55-2942] | 9753.99 [7779.69-12277.81] | 5724.18 [4523.5-7229.47] | 10331.73 [8136.23-13070.42] | -0.05 [-0.19 to 0.08] |
| Eswatini | 29.99 [22.34-40.01] | 9915.74 [7335.5-13295] | 72.89 [50.28-103.01] | 11738.94 [8037.84-16704.54] | 0.33 [0.24 to 0.43] |
| Zimbabwe | 376.16 [284.62-501.93] | 8323.46 [6236.55-11172.55] | 684.43 [486.23-966.4] | 9182.82 [6446.65-13039.23] | 0.23 [0.19 to 0.27] |
| Benin | 271 [202.21-360.99] | 11689.07 [8700.38-15593.28] | 714.84 [493.83-1002.32] | 12888.87 [8874.63-18116.58] | 0.26 [0.23 to 0.28] |
| Cameroon | 598.11 [439.73-800.6] | 12297.21 [8994.09-16510.23] | 1781.55 [1220.43-2533.61] | 13386.12 [9137.71-19092.52] | 0.22 [0.18 to 0.26] |
| Cabo Verde | 36.19 [26.4-48.85] | 12162.63 [8919.17-16399.69] | 75.58 [52.79-106.09] | 14388.33 [10021.88-20186.08] | 0.37 [0.29 to 0.46] |
| Chad | 476.1 [354.98-623.88] | 14382.41 [10693.22-18919.3] | 934.17 [654.77-1297.54] | 15232.98 [10596.96-21209.66] | 0.14 [-0.03 to 0.3] |
| Côte d'Ivoire | 410.53 [309.48-541.92] | 10415.2 [7746.36-13797.13] | 1239.54 [861.85-1729.36] | 10894.56 [7496.09-15264.53] | 0.11 [0.09 to 0.14] |
| Gambia | 61.55 [46.13-81.43] | 16497.93 [12304-21893.2] | 187.57 [131-254.03] | 17619.11 [12266.77-23926.58] | 0.1 [0.07 to 0.14] |
| Ghana | 793.69 [593.97-1060.22] | 11895.91 [8829.2-15952.3] | 2325.69 [1610.36-3266.93] | 12782.43 [8815.02-18032.06] | 0.18 [0.13 to 0.22] |
| Guinea | 448.66 [337.3-589.61] | 11239.24 [8399.69-14833.57] | 775.38 [542.16-1073.61] | 12272.76 [8529.64-17066.91] | 0.2 [0.16 to 0.24] |
| Guinea-Bissau | 49.17 [36.91-65.63] | 11434.58 [8506.8-15323.45] | 92.69 [63.21-129.74] | 12698.14 [8617.32-17851.54] | 0.29 [0.26 to 0.33] |
| Liberia | 164.7 [122.22-218.96] | 11980.7 [8836.37-15982.93] | 274.52 [192.24-378.87] | 13088.51 [9130.28-18153.78] | 0.32 [0.27 to 0.36] |
| Mali | 395.56 [298.37-525.65] | 9007.05 [6729.42-12025.59] | 886.02 [614.87-1223.53] | 9225.94 [6356.42-12782.18] | 0.03 [-0.04 to 0.11] |
| Mauritania | 114.42 [86.15-149.85] | 9970.95 [7465.51-13118.22] | 254.93 [177.46-358.47] | 10221.92 [7063.23-14410.77] | 0 [-0.07 to 0.07] |
| Niger | 321.04 [238.81-424.76] | 10991.68 [8097.63-14625.21] | 1036.14 [726.7-1448.67] | 11584.42 [8089.86-16244.03] | 0.21 [0.2 to 0.23] |
| Nigeria | 5649.01 [4355.39-7201.24] | 11229.91 [8632.65-14349.29] | 9943.51 [7603.52-12741.38] | 10453.29 [7976.12-13404.1] | -0.31 [-0.49 to -0.13] |
| Sao Tome and Principe | 6.94 [5.12-9.31] | 8797.22 [6455.75-11813.11] | 11.05 [7.7-15.81] | 9276.31 [6423.14-13313.63] | 0.17 [0.11 to 0.22] |
| Senegal | 405.47 [303.65-539.48] | 11024.58 [8208.06-14721.7] | 1038.66 [718.19-1467.88] | 11816.9 [8141.98-16713.33] | 0.12 [0.07 to 0.17] |
| Sierra Leone | 268.22 [199.29-358.44] | 11130.65 [8254.13-14892.87] | 507.08 [352.58-709.65] | 12368.8 [8558.58-17356.45] | 0.37 [0.31 to 0.44] |
| Togo | 154.15 [114.72-201.58] | 11927.4 [8816.61-15674.47] | 517.58 [363.77-724.25] | 13173.68 [9200.69-18518.81] | 0.29 [0.27 to 0.32] |
| Monaco | 6.01 [4.17-8.39] | 6505.77 [4528.16-9073.02] | 8.49 [5.6-12.35] | 6852.98 [4560.01-9909.4] | 0.03 [-0.02 to 0.08] |
| Nauru | 0.22 [0.16-0.31] | 4493.63 [3206.55-6148.59] | 0.32 [0.21-0.45] | 4939.15 [3248.4-7150.27] | 0.23 [0.2 to 0.25] |
| Niue | 0.13 [0.09-0.18] | 4706.05 [3317.5-6485.73] | 0.14 [0.09-0.19] | 4885.74 [3301.82-6922.11] | 0.04 [0.02 to 0.07] |
| Palau | 0.55 [0.39-0.75] | 4627.65 [3287.18-6335.68] | 1.33 [0.89-1.91] | 4785.86 [3192.01-6887.1] | 0.03 [0 to 0.06] |
| San Marino | 2.96 [2.04-4.13] | 6457.99 [4460.12-9008.44] | 6.27 [4.09-9.23] | 6873.64 [4527.33-10060.27] | 0.03 [-0.04 to 0.1] |
| South Sudan | 360.81 [271.23-475.03] | 12019.35 [8953.07-15926.95] | 501.39 [347.46-697.28] | 12797.81 [8803.78-17833.99] | 0.15 [0.11 to 0.19] |
| Sudan | 683.07 [514.66-920.03] | 6301.26 [4718.78-8519.32] | 1460.57 [1014.95-2072.68] | 6807.29 [4707.06-9692.47] | 0.14 [0.09 to 0.19] |

Table S4: Age-standardized disability-adjusted life year (DALY) rates (per 100,000 population) and absolute DALY counts for mental disorders among people over 60 years across 204 countries and territories, 1990-2021.

|  | 1990 | | 2021 | | EAPC(95%CI) |
| --- | --- | --- | --- | --- | --- |
|  | Numbers *10^2^ (95%UI) | ASPR(95%UI) | Numbers *10^2^ (95%UI) | ASPR(95%UI) |  |
| Mongolia | 25.49 [17.75-34.22] | 2093.52 [1456.78-2815.6] | 55.22 [38.33-77.26] | 2179.79 [1510.84-3054.65] | 0.07 [0.05 to 0.09] |
| Montenegro | 15.55 [11.08-20.65] | 2003.38 [1427.5-2663.27] | 28.64 [20.46-38.9] | 2131.53 [1521.6-2897.3] | 0.01 [-0.05 to 0.08] |
| Russian Federation | 4732.81 [3399.01-6150.91] | 1991.58 [1430.07-2589.7] | 6706.48 [4850.51-8663.97] | 2017.79 [1456.37-2608.34] | -0.16 [-0.22 to -0.1] |
| Taiwan (Province of China) | 357.32 [261.13-472.18] | 1755.06 [1280.38-2317.84] | 1046.48 [745.6-1385.4] | 1830.32 [1303.78-2422.19] | 0.09 [0.07 to 0.1] |
| Ukraine | 2400.93 [1682.05-3190.1] | 2488.65 [1744.86-3308.85] | 2607.78 [1823.27-3553.56] | 2453.08 [1713.64-3343.02] | -0.39 [-0.47 to -0.3] |
| Solomon Islands | 2.62 [1.9-3.45] | 1686.46 [1222.84-2215.94] | 6.42 [4.63-8.56] | 1733.18 [1250.62-2310.58] | 0.03 [0.01 to 0.05] |
| Romania | 681.64 [489.09-900.77] | 1854.42 [1331.21-2450.67] | 991.42 [701.22-1328.61] | 1998.22 [1413.85-2678.54] | 0.08 [0.01 to 0.14] |
| Malaysia | 218.18 [157.64-290.71] | 2050.09 [1482.08-2727.96] | 762.7 [539.26-1025.87] | 2122.71 [1501.21-2855.34] | 0 [-0.09 to 0.1] |
| Canada | 684.61 [500.53-894.18] | 1618.76 [1183.56-2114.55] | 1543.88 [1122.57-2030.81] | 1626.49 [1181.26-2140.13] | -0.17 [-0.26 to -0.08] |
| Bosnia and Herzegovina | 102.17 [73.03-135.53] | 2080.44 [1484.63-2763.09] | 175.19 [125.08-238.96] | 2064.99 [1473.7-2818.02] | -0.23 [-0.3 to -0.16] |
| Cook Islands | 0.26 [0.19-0.35] | 1754.62 [1276.86-2341.19] | 0.62 [0.44-0.83] | 1812.61 [1298.5-2439.73] | 0.06 [0.04 to 0.07] |
| Republic of Korea | 479.62 [356.34-613.11] | 1405.65 [1043.07-1798.03] | 1951.83 [1428.81-2512.85] | 1547.83 [1132.93-1993.89] | 0.42 [0.3 to 0.53] |
| Democratic People's Republic of Korea | 354.67 [256.39-471.05] | 1932.5 [1396.9-2571.37] | 755.22 [542.33-1014.23] | 1889.61 [1356.23-2540.05] | -0.12 [-0.14 to -0.11] |
| Dominican Republic | 96.71 [68.97-128.85] | 2204.2 [1572.84-2938.35] | 288.2 [198.34-398.83] | 2372.28 [1632.58-3282.75] | 0.16 [0.07 to 0.25] |
| Japan | 3084.96 [2317.02-3879.97] | 1406.15 [1055.72-1769.24] | 6227.98 [4668.44-7785.55] | 1475.87 [1107.13-1842.07] | 0.15 [0.12 to 0.18] |
| Turkmenistan | 45.97 [32.81-61.4] | 2031.62 [1447.65-2712.5] | 96.6 [66.84-128.94] | 2026.06 [1397.42-2708.68] | -0.1 [-0.16 to -0.05] |
| Democratic Republic of the Congo | 455.22 [317.88-605.19] | 2554.65 [1782.36-3410.37] | 1046.75 [724.32-1416.67] | 2643.18 [1829.37-3589.99] | 0.09 [0.06 to 0.12] |
| Grenada | 1.83 [1.32-2.43] | 1964.68 [1420.7-2604.59] | 2.88 [2.03-3.92] | 2041.92 [1439.77-2784.56] | 0.03 [-0.02 to 0.07] |
| Puerto Rico | 86.93 [62.88-114.5] | 1874.05 [1354.85-2470.06] | 175.7 [123.38-234.93] | 1921.16 [1347.74-2570.36] | 0.02 [-0.04 to 0.07] |
| Georgia | 179.02 [127.47-239.65] | 2200.97 [1567.64-2948.32] | 186.57 [126.78-255.35] | 2299.4 [1562.97-3147.27] | 0.01 [-0.03 to 0.06] |
| Palestine | 28.41 [20.15-38.07] | 2679.46 [1900.42-3589.3] | 79.88 [54.62-111.27] | 2749.9 [1884.75-3820.54] | 0.01 [-0.06 to 0.09] |
| Jamaica | 42.96 [30.62-57] | 1885.46 [1343.67-2503] | 78.05 [54.52-106.35] | 1996.29 [1396.67-2718.02] | 0.1 [0.03 to 0.16] |
| Guam | 1.63 [1.17-2.16] | 1805.25 [1294.06-2382.1] | 5.23 [3.75-7.05] | 1883.74 [1354.37-2540.97] | 0.07 [0.04 to 0.1] |
| Central African Republic | 33.12 [23.04-44.96] | 2652.2 [1847.51-3605.26] | 62.01 [42.79-86.49] | 2732.19 [1879.96-3806.49] | 0.06 [0.04 to 0.09] |
| United States Virgin Islands | 1.98 [1.44-2.63] | 1988.59 [1439.21-2641.32] | 5.25 [3.67-7.22] | 2094.01 [1463.23-2882.51] | 0.09 [0.05 to 0.13] |
| Brunei Darussalam | 1.38 [1.03-1.76] | 1269.39 [943.6-1617] | 5.41 [3.98-6.92] | 1303.47 [958.88-1673.47] | 0.05 [0.03 to 0.07] |
| Lao People's Democratic Republic | 43.16 [31.02-57.41] | 1753.52 [1261.09-2332.09] | 97.68 [69.59-132.28] | 1816.84 [1293.92-2458.57] | 0.02 [-0.02 to 0.05] |
| Tuvalu | 0.15 [0.11-0.2] | 1761.04 [1258.21-2353.97] | 0.24 [0.17-0.33] | 1801.38 [1272.4-2435.96] | 0.03 [0.01 to 0.05] |
| Antigua and Barbuda | 1.3 [0.93-1.72] | 1916.88 [1376-2539.8] | 2.7 [1.91-3.64] | 1983.21 [1404.35-2680.3] | 0.02 [-0.05 to 0.08] |
| United States of America | 7675.39 [5691.22-9799.15] | 1848.77 [1370.91-2360.85] | 15298.46 [11433.04-19568.32] | 1961.32 [1465.44-2508.76] | -0.08 [-0.19 to 0.02] |
| Hungary | 422.57 [305.3-561.66] | 2139.29 [1546.3-2845.81] | 552.43 [388.22-744.73] | 2131.71 [1497.46-2871.65] | -0.18 [-0.28 to -0.07] |
| Saint Lucia | 2.22 [1.6-2.95] | 1952.95 [1407.77-2601.5] | 6.32 [4.38-8.55] | 2079.78 [1443.94-2814.01] | 0.06 [0 to 0.12] |
| North Macedonia | 44.65 [32.37-58.73] | 1918.47 [1392.22-2523.25] | 90.42 [63.88-120.99] | 2007.98 [1418-2693.3] | -0.04 [-0.11 to 0.02] |
| American Samoa | 0.42 [0.31-0.55] | 1649.52 [1199.99-2171.21] | 0.96 [0.69-1.28] | 1650.38 [1188.69-2216.84] | -0.05 [-0.07 to -0.02] |
| Northern Mariana Islands | 0.25 [0.18-0.33] | 1667.45 [1211.92-2200.15] | 1.12 [0.8-1.51] | 1764.66 [1262.92-2380.35] | 0.09 [0.06 to 0.12] |
| Saint Vincent and the Grenadines | 1.82 [1.3-2.42] | 1956.8 [1400.03-2615.21] | 3.89 [2.72-5.25] | 2068.19 [1446.09-2792.96] | 0.08 [0.03 to 0.13] |
| Australia | 509.44 [370.86-660.2] | 1976.56 [1438.53-2562.07] | 1136.93 [832.7-1493.4] | 1980.03 [1448.83-2600.93] | 0.04 [0 to 0.08] |
| United Republic of Tanzania | 341.38 [239.84-460.34] | 2671.32 [1875.02-3608.82] | 782.29 [532.55-1070.35] | 2749.45 [1869.53-3766.82] | 0.03 [0 to 0.05] |
| New Zealand | 99.37 [72.96-127.69] | 1927.05 [1414.65-2477.51] | 204.65 [149.25-265.31] | 1877.4 [1369.76-2435.29] | -0.03 [-0.08 to 0.02] |
| Syrian Arab Republic | 122.32 [87.71-162.97] | 1983.52 [1423.51-2639.67] | 343.14 [240.94-469.79] | 2070.86 [1453.29-2833.23] | 0.08 [0.03 to 0.12] |
| Barbados | 7.42 [5.35-9.84] | 1980.61 [1425.48-2626.27] | 14.53 [10.35-19.77] | 2080.2 [1481.02-2830.68] | 0.07 [0.02 to 0.12] |
| Republic of Moldova | 131.42 [93.67-175.59] | 2304.91 [1643.53-3079.78] | 185.77 [129.75-249.85] | 2285.48 [1597.36-3071.95] | -0.18 [-0.25 to -0.12] |
| Burkina Faso | 128.96 [91.31-172.87] | 2523.92 [1783.07-3390.79] | 271.95 [188.26-379.95] | 2605.38 [1800.86-3650.35] | 0.08 [0.04 to 0.13] |
| Belize | 2.2 [1.58-2.92] | 1929.95 [1387.62-2557.7] | 7.16 [5.12-9.56] | 2037.6 [1457.42-2720.5] | 0.08 [0.03 to 0.13] |
| Ireland | 120.44 [86.47-159.49] | 2248.59 [1615.32-2978.03] | 240.75 [170.8-325.99] | 2396.95 [1699.32-3248] | 0.29 [0.25 to 0.33] |
| United Arab Emirates | 6.71 [4.79-8.9] | 1883.06 [1345.83-2493.98] | 70.64 [49.88-93] | 1875.33 [1329.61-2465.37] | -0.12 [-0.16 to -0.08] |
| Tokelau | 0.03 [0.02-0.05] | 1744.93 [1262.3-2330.87] | 0.03 [0.02-0.05] | 1795.03 [1287.35-2425.24] | 0.04 [0.03 to 0.06] |
| Bermuda | 1.66 [1.2-2.21] | 2117.05 [1530.43-2816.63] | 3.82 [2.63-5.19] | 2130.47 [1468.31-2891.58] | -0.1 [-0.15 to -0.06] |
| Saint Kitts and Nevis | 1.18 [0.82-1.59] | 2250.26 [1562.58-3034.93] | 2.18 [1.52-2.99] | 2350.87 [1639.43-3226.61] | 0.04 [0 to 0.08] |
| Greenland | 0.72 [0.52-0.95] | 1906.96 [1378.36-2498.93] | 1.86 [1.33-2.51] | 1974.18 [1411.62-2652.85] | -0.03 [-0.07 to 0.01] |
| China | 18401.51 [13638.89-23843.71] | 1819.07 [1345.37-2359.73] | 53897.67 [39939.94-69965.32] | 1993.71 [1475.42-2589.75] | 0.09 [0.02 to 0.16] |
| Cambodia | 92.88 [66.8-123.03] | 1780.49 [1281.44-2358.99] | 279.33 [197.76-376.67] | 1848.08 [1307.95-2489.17] | 0.01 [-0.02 to 0.04] |
| Indonesia | 1731.94 [1275.39-2230.17] | 1538.94 [1130.63-1981.37] | 4758.51 [3491.14-6183.33] | 1624.62 [1190.03-2107.79] | 0.07 [0.03 to 0.11] |
| Maldives | 1.91 [1.38-2.54] | 1874.75 [1354.81-2482.22] | 6.88 [4.92-9.18] | 1890.32 [1357.87-2526.15] | -0.09 [-0.12 to -0.05] |
| Myanmar | 417.24 [298.47-551.26] | 1484.25 [1064.49-1957.36] | 954.42 [674.93-1279.57] | 1579.58 [1117.57-2116.17] | 0.1 [0.06 to 0.15] |
| Philippines | 642 [466.7-835.79] | 1916.56 [1391.52-2494.58] | 1908.59 [1393.95-2498.77] | 1927.32 [1405.97-2520.84] | -0.09 [-0.14 to -0.05] |
| Sri Lanka | 217.23 [156.84-283.95] | 1707.21 [1232.84-2230.8] | 618.17 [445.85-830.86] | 1703.21 [1228.7-2289.79] | -0.15 [-0.2 to -0.09] |
| Thailand | 676.9 [487.56-896.24] | 1655.85 [1191.99-2189.4] | 2582.59 [1857.46-3476.09] | 1800.48 [1295.45-2420.63] | 0.25 [0.22 to 0.27] |
| Timor-Leste | 4.71 [3.38-6.23] | 1665.69 [1195.45-2199.28] | 18.6 [13-24.79] | 1697.16 [1186.41-2264.93] | 0 [-0.03 to 0.03] |
| Viet Nam | 719.92 [525.57-940.31] | 1418.44 [1035.28-1853.52] | 1824.48 [1332.02-2447.6] | 1462.8 [1067.77-1960.35] | 0.01 [-0.04 to 0.07] |
| Fiji | 6.38 [4.64-8.45] | 1684.19 [1223.69-2226.07] | 16.6 [11.98-22.09] | 1753.99 [1263.74-2335.52] | 0.05 [0.03 to 0.08] |
| Kiribati | 0.72 [0.52-0.95] | 1693.01 [1221.32-2246.96] | 1.46 [1.06-1.94] | 1720.16 [1244.05-2290.81] | -0.01 [-0.04 to 0.01] |
| Marshall Islands | 0.32 [0.23-0.42] | 1682.85 [1214.65-2224.77] | 0.68 [0.49-0.91] | 1681.71 [1207.97-2250.75] | -0.07 [-0.09 to -0.05] |
| Micronesia (Federated States of) | 1.02 [0.73-1.36] | 1700.83 [1220.28-2271.72] | 1.57 [1.13-2.13] | 1724.26 [1233.36-2328.36] | -0.02 [-0.04 to 0] |
| Papua New Guinea | 35.13 [25.58-46.35] | 1695.67 [1233.98-2235.72] | 92.47 [65.61-125.31] | 1711.22 [1216-2315.23] | -0.01 [-0.03 to 0.01] |
| Samoa | 1.76 [1.29-2.32] | 1702.19 [1241.88-2245.06] | 2.95 [2.11-3.96] | 1703.49 [1216.21-2292.47] | -0.06 [-0.08 to -0.04] |
| Tonga | 1.11 [0.8-1.49] | 1650.17 [1191.62-2200.95] | 1.63 [1.18-2.19] | 1686.86 [1218.39-2266.42] | 0.02 [0 to 0.04] |
| Vanuatu | 1.21 [0.88-1.61] | 1697.18 [1227.08-2247.35] | 3.46 [2.5-4.56] | 1744.23 [1257.41-2295.98] | 0.02 [0 to 0.04] |
| Armenia | 64.62 [46.28-85.53] | 1913.59 [1369.85-2533.63] | 127.87 [90.5-175.19] | 2109.18 [1492.4-2891.02] | 0.21 [0.16 to 0.27] |
| Azerbaijan | 106.1 [75.62-141.16] | 1841.01 [1308.92-2452.22] | 244.68 [170.47-333.98] | 1913.18 [1329.62-2616.18] | 0.04 [-0.01 to 0.1] |
| Kazakhstan | 324.21 [232.85-433.08] | 2094.07 [1505.67-2794.78] | 456.33 [326.49-613.98] | 2040.38 [1457.19-2746.83] | -0.28 [-0.33 to -0.23] |
| Kyrgyzstan | 82.91 [59.48-112.05] | 2234.64 [1601.83-3017.05] | 124.2 [85.49-169.35] | 2168.87 [1490.95-2965.18] | -0.23 [-0.3 to -0.16] |
| Tajikistan | 62.2 [44.76-82.32] | 1910.37 [1374.29-2530.65] | 127.39 [91.46-172.58] | 1882.77 [1350.61-2560.91] | -0.18 [-0.24 to -0.11] |
| Uzbekistan | 280.43 [200.08-370.4] | 2031.98 [1449.41-2679.76] | 612.92 [429.21-821.13] | 1971.24 [1379.99-2646.98] | -0.2 [-0.25 to -0.14] |
| Albania | 42.47 [30.75-55.38] | 1736.43 [1255.52-2267.5] | 112.73 [79.81-152.71] | 1870.29 [1323.9-2534.35] | 0.13 [0.08 to 0.18] |
| Bulgaria | 355.2 [258.19-471.14] | 2093.03 [1521.15-2777.77] | 414.87 [292.93-559.23] | 2160.1 [1527.6-2914.01] | -0.17 [-0.25 to -0.08] |
| Croatia | 182.48 [130.66-241.75] | 2292.76 [1639.04-3036.39] | 263.54 [184.23-355.09] | 2222.61 [1553.07-2999.49] | -0.28 [-0.35 to -0.21] |
| Czechia | 394.7 [284.6-528.92] | 2141.21 [1544.49-2870.89] | 569.79 [401.28-776.2] | 2048.4 [1440.44-2792.26] | -0.32 [-0.41 to -0.24] |
| Poland | 900.97 [664.55-1156.78] | 1558.31 [1149.32-2000.28] | 1642.15 [1212.93-2112.67] | 1679.39 [1239.98-2161.3] | 0.02 [-0.07 to 0.12] |
| Serbia | 311.96 [225.92-416.93] | 2078.98 [1504.36-2783.15] | 470.2 [330.65-630.99] | 2097.1 [1474.35-2813.78] | -0.15 [-0.2 to -0.1] |
| Slovakia | 157.51 [114.72-208.12] | 1988.53 [1447.51-2626.27] | 265.85 [188.47-360.74] | 2062.32 [1461.42-2800.02] | -0.08 [-0.18 to 0.01] |
| Slovenia | 74.89 [53.43-100.18] | 2366.71 [1689.95-3163.26] | 128.42 [90.52-173.86] | 2232.28 [1572.27-3021.17] | -0.39 [-0.45 to -0.32] |
| Belarus | 404.72 [287.41-545.13] | 2362.57 [1681.28-3176.64] | 559.23 [384.18-751.98] | 2527.99 [1738.22-3396.23] | -0.02 [-0.09 to 0.05] |
| Estonia | 71.82 [50.73-97.68] | 2680.66 [1893.6-3641.94] | 89.54 [62.16-122.75] | 2554.08 [1770.95-3502.45] | -0.43 [-0.52 to -0.34] |
| Latvia | 122.07 [86.33-164.08] | 2603.05 [1842.07-3495.48] | 133.54 [92.81-179.85] | 2574.25 [1790.02-3469.86] | -0.36 [-0.48 to -0.24] |
| Lithuania | 147.89 [105.1-198.34] | 2527.08 [1796.93-3388.18] | 201.27 [140.2-281.72] | 2690.55 [1873.79-3766.4] | -0.08 [-0.16 to 0] |
| Singapore | 41.64 [30.77-53.49] | 1616.74 [1192.45-2081.13] | 164.94 [119.97-212.84] | 1464.99 [1065.28-1891.2] | -0.47 [-0.54 to -0.4] |
| Andorra | 1.61 [1.16-2.11] | 2150.09 [1548.31-2827.4] | 4.22 [2.99-5.68] | 2195.47 [1557.53-2956] | 0 [-0.05 to 0.05] |
| Austria | 321.97 [232.11-419.51] | 2081.81 [1498.98-2712.04] | 465.5 [329.04-625.56] | 2088.39 [1476.18-2808.42] | -0.11 [-0.17 to -0.06] |
| Belgium | 402.75 [292.48-525.09] | 1984.49 [1440.71-2589.32] | 604.63 [435.18-792.08] | 2077.66 [1495.59-2720.24] | 0.15 [0.07 to 0.23] |
| Cyprus | 22.21 [16.09-28.91] | 2053.56 [1486.62-2676.54] | 57.56 [40.84-77.41] | 2116.76 [1500.94-2849.47] | 0.02 [-0.02 to 0.06] |
| Denmark | 238.37 [172.08-316.62] | 2296.18 [1655.9-3051.95] | 331.11 [235.75-436.06] | 2214.93 [1576.39-2916.88] | -0.19 [-0.27 to -0.1] |
| Finland | 187.94 [136.52-245.99] | 2019.46 [1467.15-2644.37] | 327.91 [235.9-436.66] | 2051.35 [1477.29-2737.1] | 0.16 [0.06 to 0.27] |
| France | 2658.55 [1939.11-3485.41] | 2467.9 [1800.98-3237.36] | 4138.65 [2994.23-5575.79] | 2383.61 [1725.11-3213.17] | -0.06 [-0.15 to 0.02] |
| Germany | 3464.11 [2486.17-4542.2] | 2138.77 [1535.23-2805.56] | 5595.23 [3970.54-7488.62] | 2331.75 [1654.47-3120.44] | 0.31 [0.17 to 0.45] |
| Greece | 535.22 [385.14-716.13] | 2671.02 [1922.04-3575.85] | 792.75 [545.42-1078.48] | 2747.35 [1892.89-3733.62] | -0.13 [-0.27 to 0.01] |
| Iceland | 7.31 [5.29-9.57] | 1992.53 [1441.69-2605.21] | 14.94 [10.76-19.99] | 1986.34 [1431.14-2656.74] | -0.05 [-0.09 to 0] |
| Israel | 145.57 [105.78-193.16] | 2286.53 [1660.27-3036.5] | 363.2 [258.17-484.08] | 2309.66 [1640.57-3079.19] | 0.02 [-0.05 to 0.1] |
| Italy | 2743.79 [1984.08-3564.26] | 2324.69 [1682.5-3020.87] | 4091.6 [2971.81-5310.23] | 2336.22 [1699.23-3031.14] | -0.04 [-0.09 to 0.02] |
| Luxembourg | 15.61 [11.35-20.45] | 2191.33 [1593.05-2871.63] | 28.07 [20.09-37.81] | 2128.57 [1524.36-2867.25] | -0.2 [-0.26 to -0.15] |
| Malta | 11.54 [8.3-15.06] | 2095.49 [1508.14-2735.5] | 27.3 [19.27-36.42] | 2121.89 [1498.49-2830.92] | -0.03 [-0.07 to 0.01] |
| Netherlands | 593.52 [433.61-767.16] | 2309.16 [1686.54-2983.55] | 1066.67 [755.49-1439.77] | 2357.18 [1668.19-3182.75] | -0.04 [-0.14 to 0.06] |
| Norway | 192.86 [140.48-250.31] | 2188.63 [1594.52-2842.81] | 283.66 [205.67-367.34] | 2255.34 [1635.94-2921.81] | 0.09 [0.06 to 0.12] |
| Portugal | 544.09 [391.3-728.53] | 2904.08 [2086.61-3891.57] | 907.09 [629.03-1230.62] | 2958.24 [2050.63-4014.19] | -0.2 [-0.35 to -0.05] |
| Spain | 1639.3 [1187.65-2140.49] | 2244.47 [1625.1-2931.47] | 2870.47 [2073.94-3850.2] | 2425.64 [1748.02-3247.13] | 0.62 [0.46 to 0.78] |
| Sweden | 441.5 [324.81-571.95] | 2288.64 [1682.37-2964.95] | 610.66 [440.07-797.54] | 2333.56 [1682.39-3047.33] | 0.06 [0.02 to 0.09] |
| Switzerland | 322.73 [234.43-429.86] | 2465.79 [1791.79-3286.82] | 564.08 [404.36-758.8] | 2549.51 [1826.3-3428.82] | -0.09 [-0.25 to 0.06] |
| United Kingdom | 2432.13 [1743.39-3155.81] | 2062.96 [1480.16-2677.18] | 3666.06 [2634.88-4775.27] | 2278.42 [1637.93-2969.7] | 0.21 [0.16 to 0.25] |
| Argentina | 733.22 [533.16-952.93] | 1743.51 [1267.28-2267.05] | 1241.53 [889.89-1623.88] | 1740.73 [1247.2-2276.56] | -0.17 [-0.23 to -0.11] |
| Chile | 252.64 [184.55-328.72] | 2039.1 [1487.78-2655.13] | 662.6 [469.96-894.66] | 1994.35 [1414.6-2692.68] | -0.18 [-0.25 to -0.12] |
| Uruguay | 90.6 [66.25-117.96] | 1748.98 [1278.68-2277.88] | 132.24 [94.13-175.02] | 1900.18 [1351.43-2514.3] | 0.13 [0.09 to 0.17] |
| Bahamas | 3.42 [2.47-4.55] | 1935.3 [1395.96-2579.14] | 10.16 [7.09-13.54] | 2014.71 [1409.02-2689.32] | 0.03 [-0.03 to 0.1] |
| Cuba | 319.17 [225.52-430.94] | 2491.33 [1759.51-3367.19] | 596.27 [429.33-815.61] | 2477.6 [1783.19-3390.51] | -0.16 [-0.23 to -0.09] |
| Dominica | 1.49 [1.07-1.98] | 1934.25 [1392.95-2571.2] | 2.19 [1.54-2.96] | 1999.18 [1408.36-2710.95] | -0.01 [-0.06 to 0.04] |
| Guyana | 10.01 [7.15-13.35] | 2238.45 [1598.51-2986.2] | 19.86 [13.81-27.11] | 2471.78 [1719.33-3378.98] | 0.19 [0.14 to 0.24] |
| Haiti | 76.76 [54.46-101.77] | 1996.74 [1417.31-2651.95] | 173.3 [121.54-235.28] | 2085.58 [1463.96-2833.78] | 0.05 [0 to 0.1] |
| Suriname | 6.82 [4.89-9.12] | 2232.89 [1602.27-2981.43] | 19.58 [13.64-26.74] | 2473.29 [1724.12-3378.83] | 0.18 [0.13 to 0.23] |
| Trinidad and Tobago | 21.16 [15.01-28.16] | 2028.54 [1438.88-2702.86] | 56.62 [40.2-75.99] | 2166.2 [1536.65-2907.8] | 0.02 [-0.05 to 0.1] |
| Bolivia (Plurinational State of) | 87.39 [61.81-117.31] | 2302.95 [1628.97-3092.03] | 281.26 [194.92-386.29] | 2470.22 [1712.16-3392.07] | 0.02 [-0.06 to 0.11] |
| Ecuador | 127.8 [91.36-170.49] | 2055.13 [1469.82-2740.68] | 461.8 [324.09-608.19] | 2273.5 [1595.07-2995.61] | 0.23 [0.15 to 0.3] |
| Peru | 261.25 [185.51-344.18] | 1865.01 [1325.14-2456.56] | 801.42 [568.59-1084.53] | 1966.92 [1395.58-2662.1] | -0.03 [-0.12 to 0.06] |
| Colombia | 366.89 [266.85-483.75] | 1781.26 [1295.36-2346.42] | 1274.68 [887-1708.97] | 1823.87 [1269.7-2444.15] | -0.14 [-0.25 to -0.03] |
| Costa Rica | 41.57 [29.92-55.34] | 1982.61 [1427.45-2639.06] | 148.9 [104.19-201.07] | 2115.38 [1481.46-2856.6] | 0.12 [0.07 to 0.16] |
| El Salvador | 72.51 [51.78-96.36] | 2037.89 [1455.69-2706.82] | 160.54 [111.54-219.07] | 2109.46 [1466.43-2876.37] | 0 [-0.05 to 0.06] |
| Guatemala | 90.17 [64.16-120.6] | 2221.61 [1582.72-2972.84] | 312.21 [217.3-429.29] | 2321.28 [1614.78-3192.42] | -0.06 [-0.24 to 0.12] |
| Honduras | 48.14 [34.53-63.82] | 2026.76 [1452.86-2689.93] | 172.41 [116.86-235.05] | 2258.47 [1530.4-3083.49] | 0.23 [0.19 to 0.28] |
| Mexico | 961.37 [701.3-1238.81] | 1932.35 [1409.23-2491.11] | 3285.97 [2372.65-4259.69] | 2100.87 [1517.47-2723.96] | 0.05 [-0.03 to 0.13] |
| Nicaragua | 34.3 [24.43-45.59] | 1943.25 [1383.64-2582.93] | 121.36 [85.95-165.35] | 2074.78 [1469.4-2828.18] | 0.12 [0.07 to 0.16] |
| Panama | 34.53 [24.73-46.02] | 1941.64 [1390.54-2587.53] | 110.46 [77.49-145.81] | 2021.31 [1418.34-2667.2] | 0.03 [-0.02 to 0.08] |
| Venezuela (Bolivarian Republic of) | 232.72 [166.61-312.17] | 2053.89 [1469.99-2754.5] | 812.43 [562.63-1113.2] | 2108.97 [1460.09-2891.45] | 0.01 [-0.02 to 0.04] |
| Brazil | 2595.21 [1882.26-3392.04] | 2413.28 [1752.12-3151.81] | 8344.47 [6004.93-10892.2] | 2615.35 [1882.49-3412.84] | 0.13 [0 to 0.25] |
| Paraguay | 57.11 [41.08-76.6] | 2139.37 [1539.26-2868.56] | 167.69 [116.26-231.41] | 2318.62 [1609.46-3198.02] | 0.13 [0.08 to 0.19] |
| Algeria | 308.19 [220.35-410.41] | 2018.08 [1443.68-2688.22] | 891.11 [628.33-1200.76] | 2046.75 [1442.51-2757.02] | -0.02 [-0.05 to 0.01] |
| Bahrain | 4.16 [3.01-5.51] | 2233.82 [1616.38-2954.28] | 23.25 [16.22-30.77] | 2207.35 [1546.47-2928.39] | -0.19 [-0.24 to -0.14] |
| Egypt | 585.75 [422.24-774.55] | 1850.62 [1332.86-2443.78] | 1571.83 [1119.15-2110.76] | 2014.44 [1434.92-2703.5] | 0.15 [0.08 to 0.22] |
| Iran (Islamic Republic of) | 740.52 [529.79-974.57] | 2274.58 [1626.32-2990.29] | 2271.83 [1614.76-3005.38] | 2408.24 [1712.72-3183.09] | 0.25 [0.2 to 0.3] |
| Iraq | 167.76 [121.5-220.42] | 1838.4 [1331.12-2415.46] | 516.97 [368.86-689.01] | 1895.59 [1352.39-2525.86] | 0.06 [-0.02 to 0.13] |
| Jordan | 30.1 [21.53-39.88] | 2077.71 [1487.16-2752.56] | 170.95 [121.3-232] | 2035.62 [1444.24-2765.6] | -0.17 [-0.21 to -0.13] |
| Kuwait | 11.55 [8.38-15.2] | 1908.65 [1386.82-2506.21] | 57.59 [40.82-77.26] | 1955.03 [1386.94-2621.74] | 0.09 [0.07 to 0.12] |
| Lebanon | 61.29 [44.38-81.21] | 2253.43 [1633.55-2983.64] | 177.08 [125.72-242.48] | 2463.16 [1748.31-3374.18] | 0.17 [0.07 to 0.27] |
| Libya | 44.74 [32.03-59.33] | 2025.73 [1450.12-2685.18] | 120.48 [83.94-163.05] | 2132.94 [1488.24-2886.54] | 0.11 [0.08 to 0.14] |
| Morocco | 395.95 [281.16-530.24] | 2285.8 [1624.3-3061.51] | 1025.89 [711.31-1399.96] | 2357.66 [1639.66-3211.1] | 0 [-0.04 to 0.04] |
| Oman | 14.11 [10.22-18.67] | 1947.29 [1411.09-2574.89] | 42.85 [30.48-58.05] | 2092.7 [1487.28-2829.09] | 0.19 [0.16 to 0.22] |
| Qatar | 1.79 [1.3-2.36] | 1981.99 [1441.19-2604.64] | 16.88 [12.07-22.41] | 2001.15 [1426.85-2656.48] | -0.03 [-0.06 to 0] |
| Saudi Arabia | 119.66 [87.05-157.41] | 1884.36 [1372.81-2478.04] | 378.98 [271.18-509.58] | 1996.85 [1430.7-2680.53] | 0.19 [0.16 to 0.21] |
| Tunisia | 154.52 [109.42-204.39] | 2483.56 [1759.77-3292.65] | 460.16 [317.76-635.52] | 2664.08 [1839.45-3679.77] | 0.05 [-0.01 to 0.11] |
| Turkey | 784.11 [568.55-1035.47] | 1892.65 [1369.93-2502.11] | 2302.03 [1607.04-3112.23] | 1922.31 [1343.06-2598.65] | 0.08 [0.01 to 0.15] |
| Yemen | 118.62 [85.01-158.51] | 2065.54 [1480.87-2763.22] | 342.75 [239.11-462.98] | 2124.43 [1483.36-2871.22] | 0.07 [0.05 to 0.09] |
| Afghanistan | 177.06 [127.13-234.27] | 2029.92 [1457.24-2686.06] | 171.49 [122.76-230.57] | 2143.62 [1530.41-2885.32] | 0.1 [0.07 to 0.13] |
| Bangladesh | 1272.67 [902.04-1679.6] | 2349.43 [1665.75-3100.47] | 4366.79 [2987.98-6040.74] | 2550.2 [1744.25-3531.87] | 0.25 [0.19 to 0.3] |
| Bhutan | 6.72 [4.79-8.97] | 2468.39 [1759.17-3300.62] | 17.69 [12.3-24.22] | 2423.24 [1685.53-3318.16] | -0.08 [-0.12 to -0.04] |
| India | 11836.3 [8622.64-15283.13] | 2208.06 [1610.91-2849.42] | 34057.42 [24949.03-44058.89] | 2302.14 [1686.84-2979.9] | -0.14 [-0.28 to -0.01] |
| Nepal | 278.34 [194.84-369.32] | 2578.64 [1807.34-3435.82] | 841.18 [568.38-1167.44] | 2892.57 [1953.8-4023.76] | 0.28 [0.14 to 0.43] |
| Pakistan | 1613.79 [1138.35-2124.94] | 2419.54 [1707.27-3189.09] | 3314.86 [2331.74-4392.23] | 2403.52 [1694.55-3184.39] | -0.12 [-0.18 to -0.06] |
| Angola | 110.53 [76.28-149.91] | 2675.81 [1846.8-3634.88] | 348.85 [238.1-486.49] | 2769.84 [1886.39-3862.79] | 0.05 [0.02 to 0.08] |
| Congo | 33.67 [23.55-45.82] | 2708.66 [1895.02-3694.1] | 77.46 [53.41-108.39] | 2720.08 [1865.12-3817.02] | -0.06 [-0.09 to -0.03] |
| Equatorial Guinea | 5.7 [4-7.69] | 2627.36 [1840.04-3546.5] | 14.7 [10.19-20.7] | 2766.9 [1917.48-3902.8] | 0.15 [0.12 to 0.18] |
| Gabon | 19.1 [13.35-25.82] | 2721.02 [1902.33-3684.78] | 33.83 [23.33-47.57] | 2790.33 [1922.89-3931.44] | -0.01 [-0.05 to 0.02] |
| Burundi | 79.25 [54.84-107] | 2936.81 [2032.15-3971.62] | 150.92 [103.11-209.28] | 2757.7 [1885.58-3826.15] | -0.38 [-0.45 to -0.32] |
| Comoros | 5.7 [3.99-7.67] | 2491.58 [1741.18-3360.78] | 14.58 [10.05-20.23] | 2583.35 [1778.09-3591.33] | 0.05 [-0.02 to 0.11] |
| Djibouti | 3.5 [2.45-4.65] | 2543.67 [1775.45-3395.84] | 17.81 [12.31-24.61] | 2623.91 [1815.25-3641.01] | 0.04 [0.02 to 0.07] |
| Eritrea | 30.18 [20.75-41.19] | 2804.91 [1935.9-3820.12] | 81.47 [54.06-113] | 2832.67 [1876.66-3921.08] | -0.04 [-0.06 to -0.01] |
| Ethiopia | 607.86 [422.23-810.28] | 2715.53 [1886.44-3626.61] | 1335.94 [946.12-1766.02] | 2759.09 [1952.42-3654.35] | -0.03 [-0.11 to 0.05] |
| Kenya | 267.84 [190.48-349.92] | 2841.67 [2019.85-3719.81] | 747.29 [532.77-971.21] | 2856.62 [2037.59-3723.38] | -0.11 [-0.17 to -0.06] |
| Madagascar | 160.15 [113.46-214.01] | 2708.32 [1914.49-3622.95] | 339.88 [233.23-467.46] | 2822.46 [1931.28-3903.14] | 0.05 [0.02 to 0.09] |
| Malawi | 109.61 [78.2-146.44] | 2429.69 [1731.46-3256.78] | 210.13 [144.91-280.81] | 2515.1 [1733.42-3370.55] | 0 [-0.05 to 0.05] |
| Mauritius | 18.6 [13.38-24.65] | 2050.26 [1476.45-2715.72] | 50.49 [36.29-67.03] | 2027.7 [1458.36-2696.34] | -0.2 [-0.3 to -0.1] |
| Mozambique | 177.24 [124.8-239.64] | 2615.29 [1836.3-3540.23] | 354.03 [240.19-482.94] | 2880.19 [1953.68-3936.61] | 0.24 [0.22 to 0.27] |
| Rwanda | 100.33 [69.41-135.41] | 3051.58 [2114.66-4125.91] | 219.43 [147.12-309.98] | 2974 [1994.31-4221.61] | -0.26 [-0.31 to -0.2] |
| Seychelles | 1.24 [0.89-1.64] | 1708.06 [1228.67-2265.86] | 2.54 [1.83-3.44] | 1742.66 [1248.66-2354.67] | -0.07 [-0.12 to -0.03] |
| Somalia | 60.39 [41.72-81.12] | 2653.91 [1837.19-3565.32] | 187.82 [126.13-259.82] | 2835.77 [1903.14-3931.99] | 0.08 [0.05 to 0.12] |
| Uganda | 264.33 [183.7-355.37] | 3569.03 [2476.93-4809.65] | 604.87 [405.6-846.23] | 3762.62 [2519.99-5271.42] | -0.02 [-0.1 to 0.06] |
| Zambia | 74.27 [52.52-99.01] | 2367.49 [1672.39-3162.5] | 186.46 [128.54-258.96] | 2544.06 [1753.68-3536.51] | 0.15 [0.09 to 0.22] |
| Botswana | 14.88 [10.49-19.83] | 2250.73 [1582.78-3008.67] | 40.99 [28.44-56.76] | 2436.35 [1685.35-3379.47] | 0.11 [0.08 to 0.15] |
| Lesotho | 29.25 [20.3-39.72] | 2915.51 [2021.01-3970.61] | 39.63 [26.97-54.7] | 3067.08 [2080.85-4235.36] | 0.08 [0.03 to 0.14] |
| Namibia | 16.36 [11.73-21.79] | 2072.86 [1480.67-2770.16] | 35.38 [24.69-48.34] | 2232.34 [1553.31-3051.05] | 0.03 [-0.03 to 0.09] |
| South Africa | 568.47 [412.3-742.61] | 2323.52 [1684.77-3037.11] | 1369.62 [994.34-1782.09] | 2420.93 [1757.42-3152.52] | -0.04 [-0.12 to 0.04] |
| Eswatini | 7.2 [5.07-9.68] | 2304.46 [1622.56-3101.93] | 16.36 [11.35-22.55] | 2560.47 [1769.84-3530.6] | 0.17 [0.1 to 0.23] |
| Zimbabwe | 96.33 [68.36-127.3] | 2038.85 [1445.05-2703.97] | 168.23 [118.65-229.18] | 2154.45 [1516.34-2949.4] | 0.1 [0.08 to 0.13] |
| Benin | 56.18 [39.88-75.09] | 2392.8 [1697.25-3203.49] | 147.08 [102.17-204.21] | 2594.75 [1799.05-3609.24] | 0.22 [0.2 to 0.24] |
| Cameroon | 125.24 [88.42-167.31] | 2487 [1752.94-3324.66] | 365.11 [245.82-500.33] | 2661.77 [1788.55-3654.37] | 0.18 [0.16 to 0.2] |
| Cabo Verde | 7.46 [5.15-10.02] | 2530.26 [1750.48-3395.18] | 15.19 [10.44-21.16] | 2841.39 [1956.78-3950.74] | 0.26 [0.2 to 0.32] |
| Chad | 92.34 [63.84-124.37] | 2762.84 [1911.16-3735.79] | 182.1 [125.6-253.01] | 2896.16 [1986.81-4031.54] | 0.13 [0.02 to 0.24] |
| Côte d'Ivoire | 91.76 [65.45-121.87] | 2205.71 [1572.25-2936.98] | 276.62 [192.6-378.05] | 2337.29 [1617.67-3206.69] | 0.15 [0.13 to 0.16] |
| Gambia | 11.78 [8.23-15.97] | 3077.38 [2144.73-4182.67] | 34.94 [23.27-47.58] | 3231.51 [2151.03-4410.61] | 0.07 [0.05 to 0.1] |
| Ghana | 168.87 [119.29-224.18] | 2445.03 [1723.76-3259.92] | 486.16 [327.14-675.17] | 2593.25 [1743.25-3602.55] | 0.15 [0.12 to 0.18] |
| Guinea | 96.39 [68.24-128.91] | 2364.14 [1670.4-3171.13] | 162.95 [113.04-223.62] | 2526.22 [1748.55-3475.29] | 0.15 [0.13 to 0.18] |
| Guinea-Bissau | 10.5 [7.35-14.11] | 2355.25 [1646.59-3166.39] | 19.4 [13.5-27.02] | 2555.82 [1771.35-3566.15] | 0.22 [0.2 to 0.24] |
| Liberia | 33.93 [23.9-45.53] | 2422.02 [1705.04-3258.82] | 55.84 [38.42-76.53] | 2592.37 [1780.81-3561.1] | 0.25 [0.22 to 0.29] |
| Mali | 92.61 [66.13-122.69] | 2017.67 [1437.05-2681.02] | 206.77 [143.11-285.44] | 2077.23 [1436.68-2878.68] | 0.07 [0.02 to 0.12] |
| Mauritania | 25.42 [17.91-33.77] | 2187.38 [1541.29-2915.91] | 57.5 [39.5-78.68] | 2254.12 [1544.5-3090.37] | 0.05 [0 to 0.1] |
| Niger | 70.48 [49.79-94.59] | 2298.06 [1618.85-3093.67] | 223.06 [157.56-305.34] | 2401.4 [1692.28-3300.04] | 0.19 [0.18 to 0.2] |
| Nigeria | 1213.81 [862.07-1599.32] | 2353.4 [1670.25-3107.44] | 2256.19 [1609.21-2966.62] | 2289.61 [1629.64-3017.63] | -0.11 [-0.2 to -0.02] |
| Sao Tome and Principe | 1.67 [1.18-2.21] | 2062.82 [1450.77-2734.73] | 2.63 [1.83-3.55] | 2131.93 [1483.39-2893.85] | 0.1 [0.07 to 0.14] |
| Senegal | 87.24 [60.87-116.45] | 2314.31 [1613.18-3095.97] | 219.44 [149.82-301.14] | 2441.13 [1664.56-3358.28] | 0.1 [0.06 to 0.13] |
| Sierra Leone | 57.43 [40.68-76.52] | 2352.44 [1665.03-3141.57] | 106.19 [73.14-148.18] | 2538.13 [1746.42-3548.34] | 0.28 [0.24 to 0.31] |
| Togo | 32.88 [23.14-43.93] | 2468.23 [1735.58-3307.29] | 108.43 [75.47-151.12] | 2664.51 [1850.94-3731.57] | 0.23 [0.22 to 0.25] |
| Monaco | 2.08 [1.49-2.79] | 2287.96 [1633.94-3071.91] | 2.89 [2.01-3.92] | 2374.86 [1653.9-3206.79] | 0.03 [-0.01 to 0.06] |
| Nauru | 0.09 [0.06-0.12] | 1725.02 [1245.08-2301.46] | 0.12 [0.08-0.16] | 1801.11 [1267.99-2450.75] | 0.1 [0.07 to 0.12] |
| Niue | 0.05 [0.04-0.07] | 1776.69 [1272.36-2371.61] | 0.05 [0.04-0.07] | 1809.66 [1298.44-2437.25] | 0.01 [-0.01 to 0.03] |
| Palau | 0.21 [0.15-0.28] | 1755.01 [1268.19-2343] | 0.51 [0.36-0.69] | 1776.63 [1262.69-2404.21] | -0.01 [-0.03 to 0] |
| San Marino | 1.03 [0.73-1.37] | 2254.54 [1611.75-3008.14] | 2.1 [1.46-2.89] | 2360.05 [1637.16-3240.63] | 0.04 [-0.01 to 0.09] |
| South Sudan | 78.55 [55.36-105.1] | 2558.92 [1802.85-3433.96] | 108.89 [74.1-149.85] | 2692.59 [1829.56-3703.72] | 0.13 [0.11 to 0.16] |
| Sudan | 212.8 [153.01-280.69] | 1940.15 [1394.06-2560.97] | 438.57 [313.84-590.28] | 2024.93 [1448.51-2726.66] | 0.06 [0.03 to 0.1] |
